# Griffin: A Tool for Symbolic Inference of Synchronous Boolean Molecular Networks

**DOI:** 10.3389/fgene.2018.00039

**Published:** 2018-03-06

**Authors:** Stalin Muñoz, Miguel Carrillo, Eugenio Azpeitia, David A. Rosenblueth

**Affiliations:** ^1^Instituto de Investigaciones en Matemáticas Aplicadas y en Sistemas, Universidad Nacional Autónoma de México, Mexico City, Mexico; ^2^Facultad de Ingeniería, Universidad Nacional Autónoma de México, Mexico City, Mexico; ^3^Maestría en Ciencias de la Complejidad, Universidad Autónoma de la Ciudad de México, Mexico City, Mexico; ^4^Institut National de Recherche en Informatique et en Automatique Project-Team Virtual Plants, Inria, CIRAD, INRA, Montpellier, France; ^5^Department of Evolutionary Biology and Environmental Studies, University of Zurich, Zurich, Switzerland; ^6^Centro de Ciencias de la Complejidad, Universidad Nacional Autónoma de Mexico, Mexico City, Mexico

**Keywords:** molecular networks, Boolean networks, model inference, Boolean satisfiability problem, clause learning, biological constraints, attractors

## Abstract

Boolean networks are important models of biochemical systems, located at the high end of the abstraction spectrum. A number of Boolean gene networks have been inferred following essentially the same method. Such a method first considers experimental data for a typically underdetermined “regulation” graph. Next, Boolean networks are inferred by using biological constraints to narrow the search space, such as a desired set of (fixed-point or cyclic) attractors. We describe *Griffin*, a computer tool enhancing this method. *Griffin* incorporates a number of well-established algorithms, such as Dubrova and Teslenko's algorithm for finding attractors in synchronous Boolean networks. In addition, a formal definition of regulation allows *Griffin* to employ “symbolic” techniques, able to represent both large sets of network states and Boolean constraints. We observe that when the set of attractors is required to be an *exact* set, prohibiting additional attractors, a naive Boolean coding of this constraint may be unfeasible. Such cases may be intractable even with symbolic methods, as the number of Boolean constraints may be astronomically large. To overcome this problem, we employ an Artificial Intelligence technique known as “clause learning” considerably increasing *Griffin*'s scalability. Without clause learning only toy examples prohibiting additional attractors are solvable: only one out of seven queries reported here is answered. With clause learning, by contrast, all seven queries are answered. We illustrate *Griffin* with three case studies drawn from the *Arabidopsis thaliana* literature. *Griffin* is available at: http://turing.iimas.unam.mx/griffin.

## 1. Introduction

Synchronous Boolean networks (Kauffman, 1969), as simple as they are, can encode meaningful biological information (Huang, 1999; Bornholdt, 2001, 2005, 2008; Fisher and Henzinger, 2007). Hence, such models have emerged as valuable candidates for representing dynamics of molecular networks. At the same time, the *inference* of network dynamics from experimental data (sometimes called the “inverse problem”) has become increasingly relevant with the advent of high-throughput technologies. Because of their simplicity, synchronous Boolean networks could become excellent network-inference models. Nevertheless, the problem of synchronous Boolean-network inference is neglected, as efforts in Boolean-network research have mainly been devoted to *analysis*. Our objective is to present and illustrate the practical use of *Griffin*, a computer tool for the inference of synchronous Boolean networks.

### 1.1. Inference of boolean networks

The inference of Boolean networks from biological data is at present coming of age. On the one hand, a methodology that has been employed to infer a number of Boolean gene regulatory networks has established itself. This methodology follows the next three steps. The first one is determining the set of genes regulating each gene, in addition to the “sign” of each regulation based on experimental results. Second, a number of constraints are considered. These constraints can be, for example, (a) a set of input-output pairs representing time-series data (Liang et al., 1998; Akutsu et al., 1999; Lähdesmäki et al., 2003; Chueh and Lu, 2012; Haider and Pal, 2012; Berestovsky and Nakhleh, 2013; Han et al., 2014; Barman and Kwon, 2017), (b) a desired set of fixed-point attractors (Albert and Othmer, 2003; Li et al., 2004; Pal et al., 2005; Mendoza, 2006; Davidich and Bornholdt, 2008; Tarissan et al., 2008; La Rota et al., 2011; Azpeitia et al., 2013, 2014), or (c) a set of temporal-logic constraints (Bernot et al., 2004; Chabrier-Rivier et al., 2004; Fages et al., 2004; Calzone et al., 2006a,b; Mateus et al., 2007; Khalis et al., 2009; Richard et al., 2012). Third, search is performed to find Boolean networks consistent with the available information.

On the other hand, there are now many methods and algorithms for the analysis of Boolean networks that have demonstrated their effectiveness. We noticed that some of these algorithms can also be used as auxiliary techniques for the inference of network dynamics. In particular, we have selected Dubrova and Teslenko's (2011) algorithm for finding cyclic attractors in synchronous Boolean networks, Garg et al.'s (2008) method for finding basins of attraction, and Naldi et al.'s (2007) formula for finding fixed-point attractors. (We use the terms “steady state dynamics” and “fixed-point attractors” interchangeably.) *Griffin* has combined these algorithms with novel techniques, offering a tool automating many steps of the inference of molecular network dynamics.

### 1.2. Synchronous boolean networks

Boolean networks have variables representing the genes, mRNA, proteins, or any other type of molecule included in the network. Variables have only two values, and time is discrete. A *state* is a tuple (i.e., ordered list) of values, one for each variable. A Boolean network consists of a (finite) set of states together with a “transition” relation (sometimes also called “update” or “successor” relation).

In asynchronous Boolean networks, states may have more than one successor. In synchronous Boolean networks, by contrast, states have exactly one successor. Previous studies suggest that having multiple successors can provide a closer description of the biological phenomena and can eliminate dynamical artifacts (Garg et al., 2008). There may be advantages, nevertheless, in considering synchronous networks.

1. A first advantage appears if the properties of interest in asynchronous networks are preserved in synchronous networks. Large models treated as synchronous networks may be easier to analyze than if treated with more detailed models. It is straightforward to see that the set of fixed-point attractors of an asynchronous network is the same as in a corresponding synchronous network (Gershenson, 2002; Bornholdt, 2008; Garg et al., 2008; Saadatpour et al., 2010). The reason is that the successor of a state that is a fixed-point attractor is itself, so that the Hamming distance between such an attractor and its successor is zero. Since a synchronous network only differs from its corresponding asynchronous one in transitions with a Hamming distance greater than one, the same self-loops will be present in both. Another property that is preserved is the set of cycles in which the Hamming distance between a state and its successor is one. Yet another one is the set of “trap subspaces.” A trap subspace is a set of states, all of which have the same values for some variables, which is closed under update. Trap spaces are important in that each trap space contains at least one attractor (Klarner et al., 2014).
  It may be the case, however, that the properties of interest are not preserved by this reduction, so that asynchronous networks will be preferable. For some problems, therefore, synchronous networks may be more appropriate, and vice versa.
2. Synchronous Boolean networks can be valuable for methods employing non-Boolean models (such as discrete, stochastic, or differential) (Fages and Soliman, 2008): A synchronous Boolean network that does not have an expected set of fixed-point attractors, for example, reflects the fact that there is something basically wrong in more detailed models of such a network. Hence, basic errors can be detected before using more precise models. A reason for preferring to detect such errors in a coarse model is that even in Boolean models a combinatorial explosion can readily occur, so that in finer corresponding models such an explosion is even more likely to occur.

The rest of this article only deals with synchronous Boolean networks. For simplicity, we will sometimes drop the “synchronous” adjective and refer to such networks simply as “Boolean.”

### 1.3. Outline of *Griffin*'s methodology

In a nutshell, *Griffin*'s methodology is the following. *Griffin* has as input a (generalization of a) “regulation” graph (sometimes also called “interaction” or “influence” graph), in addition to biological constraints, such as an expected set of either fixed-point or cyclic attractors of both the wild-type and of mutants of an organism. The output is typically a set of Boolean networks satisfying such constraints.

Because in *Griffin* the regulation graph plays a prominent role, the definition of a regulation is fundamental. *Griffin* uses the definition of Naldi et al. (2007), Richard et al. (2012), and Mori and Mochizuki (2017).

A regulation graph is normally an *underdetermined* specification of a Boolean network because even with the formal definition of regulation and sometimes also due to lack of information the regulation graph may be satisfied by many Boolean networks.

*Griffin* then combines the regulation graph with the biological constraints, to reduce the number of possible networks. This is essentially done by constructing a typically large Boolean formula representing: (1) a formal definition of the regulation graph and (2) the biological constraints. This formula is then given to a “SAT solver.” These solvers employ algorithms finding value assignments to the variables occurring in the formula that make such a formula true. Each such assignment represents a possible network.

Notice that both the regulation graph and many of the biological constraints rely on biological information. Thus, *Griffin* mainly produces biological meaningful solutions (i.e., networks that are coherent with the available experimental information).

*Griffin*'s methodology required the development of multiple innovations, including the generalization of the concept of a regulation graph that we call R-graph, the gradual application of biological constraints at runtime using clause learning, and the handling of both mutations and partially known fixed-point attractors. Some of *Griffin*'s innovations will be described below.

### 1.4. Symbolic techniques

*Griffin* relies heavily on “symbolic” methods. Such methods do not represent sets explicitly, but rather codified as Boolean expressions. This allows for the representation of large sets of states, as well as the pruning of large fragments of the search space. It is important to observe that numerous off-the-shelf symbolic computational tools exist.

### 1.5. Background of *Griffin*

The original prototype of *Griffin* appeared in Rosenblueth et al. (2014), where the authors presented a formal framework for the inference of Boolean networks from a standard regulation graph by direct application of a SAT solver to Boolean formulas codifying regulations, fixed-point attractors, and single-point mutations.

Examples illustrating the use of *Griffin* already appeared in: Rosenblueth et al. (2014), employing sets of desired fixed-points for mutations; Weinstein et al. (2015), looking for interactions that are necessary for the existence of a cyclic attractor; García-Gómez et al. (2017), verifying if a set of regulations was enough to obtain a desired set of expected attractors; and Azpeitia et al. (2017), making extensive use of hypotheses and partially defined sets of attractors for multi-point mutations.

Features illustrated in previous articles mentioning *Griffin* have been left out from this work. We demonstrate in this article *Griffin*'s new functionalities through three cases studies in section 2.2.

### 1.6. Organization

The rest of this article is organized as follows. The section “sect:results” has two parts: first we give a description of *Griffin*, and then we turn our attention to three case studies. Next follows the section “sect:discussion,” relating our work with other approaches and summarizing our results. Section 4 gives formal definitions appearing in the pseudo-code of the algorithms. Finally, the Supplementary Material includes all query files for the case studies, and a detailed explanation of the syntax used to formulate partially defined fixed-point constraints.

## 2. Results

This section first gives an account of *Griffin*, and then presents three case studies.

### 2.1. Griffin

#### 2.1.1. R-graphs

A first contribution of *Griffin* is a generalization of the ordinary regulation graph that we call “R-graph” (for “regulation”). In an R-graph, vertices are, as in an ordinary regulation graph, molecular species. However, instead of having edges denoting only two kinds of regulation (positive or negative), an R-graph has 16 kinds of labels for edges, allowing the user to express a range of nuances of what is known about a regulation. Table 1 shows these 16 possible labels. In addition to having the ordinary positive and negative regulations (first two lines), we have ambiguous regulations (third line). Ambiguous regulations are regulations that depending on the “context” are negative or positive. As in ordinary regulation graphs, it is also possible to indicate the fact that we know that there is no regulation from one molecule to another (fourth line). We, however, view labels of edges as Boolean functions over the set of positive and negative regulations. This allows us to represent additional possibilities in a uniform manner. For example, we can represent the contingency in which we know that in certain contexts a regulation is positive, but we do not know whether or not there are other contexts where such a regulation is negative. This case would correspond to the fifth line (“Mandatory, positive, possibly ambiguous”).

**Table 1:** Interpretation of the 16 R-regulations between vertices *u* and *v* for an R-graph.

**Depiction**	**Meaning**	**Acronym**	**Formula**
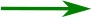	Mandatory, positive, unambiguous	MPU	*R*^+^∧¬*R*^−^
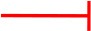	Mandatory, negative, unambiguous	MNU	¬*R*^+^∧*R*^−^
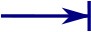	Mandatory, ambiguous	MA	*R*^+^∧*R*^−^
	No regulation	NR	¬*R*^+^∧¬*R*^−^
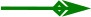	Mandatory, positive, possibly ambiguous	MPPA	*R*^+^
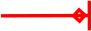	Mandatory, negative, possibly ambiguous	MNPA	*R*^−^
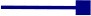	Mandatory, unknown sign, unambiguous	MUSU	¬(*R*^+^ ↔ *R*^−^)
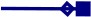	Mandatory, unknown sign, possibly ambiguous	MUSPA	*R*^+^∨*R*^−^
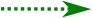	Optional, positive, unambiguous	OPU	¬*R*^−^
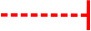	Optional, negative, unambiguous	ONU	¬*R*^+^
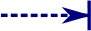	Optional, ambiguous	OA	(*R*^+^↔*R*^−^)
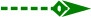	Optional, positive, possibly ambiguous	OPPA	*R*^+^∨¬*R*^−^
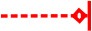	Optional, negative, possibly ambiguous	ONPA	¬*R*^+^∨*R*^−^
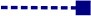	Optional, unknown sign, unambiguous	OUSU	¬(*R*^+^∧*R*^−^)
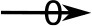	Contradiction	False	0
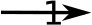	Tautology	True	1

*Each R-regulation is denoted by an acronym. Leaving out trivial regulations false, NR, and true, R-regulations can be classified as optional or mandatory. In the case of no regulation (NR), edges are not depicted. The last column shows the constraint added by Griffin in each case (R^+^ denotes a positive regulation from u to v for the Boolean network f (R^+^(u, f, v)), R^−^ a negative regulation (R^−^(u, f, v)), see definition 3)*.

Other interesting situations are the optional regulations. Such regulations can be used to test or predict putative regulations. Just as in mandatory regulations, the sign of optional regulations can vary. For instance, the first optional line (“Optional, positive, unambiguous”) represents a situation where we wish to allow for the possibility of no regulation, but in case there is one it should be positive and cannot be negative. In line 14 (last line before “Contradiction”), by contrast, we allow for the possibility of a positive regulation, a negative regulation, or no regulation.

The last column of Table 1 shows the corresponding Boolean function over positive and negative regulations (*R*^+^ and *R*^−^), defined in section 4.

We can thus represent any combination of necessity and possibility for positive and negative regulations. This generalization includes two trivial functions (Contradiction and Tautology) listed in the last two lines of Table 1.

#### 2.1.2. Meaning of a regulation

The next question we have to address is the *meaning* of a (positive or negative) “functional regulation” (i.e., *R*^+^ and *R*^−^). This meaning is important in the sense that it establishes a connection between regulation graphs (or R-graphs) and state graphs. We take the meaning proposed by Naldi et al. (2007) (and used also by Richard et al., 2012 and Mori and Mochizuki, 2017). According to this definition, a regulation is functional if it is sufficient to modify the activity of the regulated variable in a non-empty set of (molecular) contexts. A context is a specific combination of values of all other variables. Observe that it is the formal definition of regulation (Defn. 3) that allows us to define R-graphs. Some previous works (e.g., Espinosa-Soto et al., 2004) have inferred gene network dynamics manually, and without employing a formal definition of regulation. As a result, it is not clear in such inferences whether or not all possibilities of networks that are coherent with the experimental information have been explored.

**Algorithm 1:**
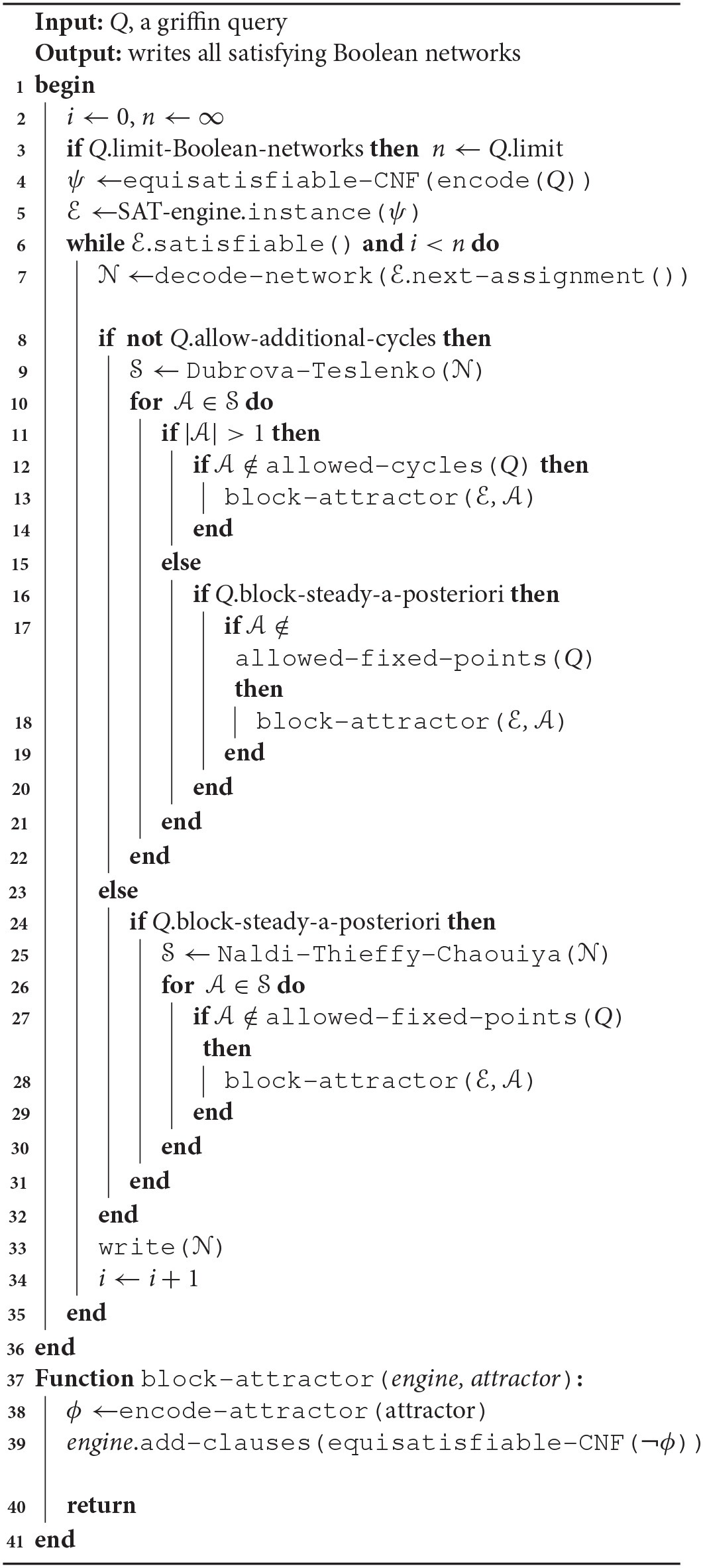
Griffin strategy for network iteration

#### 2.1.3. Tractability of boolean-network inference

Apart from an R-graph, an important input to *Griffin* can be a desired set of attractors (be they fixed-point or cyclic). Writing a Boolean formula representing a desired fixed-point, for instance, is straightforward (Rosenblueth et al., 2014). Hence, a set of such fixed-points can be represented as the conjunction (the “AND” function) of such formulas. The resulting formula, however, does not say anything about the *unwanted* fixed-points, and the solution networks might have additional fixed-points. Although such solutions, with additional fixed-points, might be acceptable, it may also be the case that we do not wish additional fixed-points. We must then explicitly express so in the Boolean formula (a “clause”), so as to block each unwanted fixed-point from solutions. This situation is analogous to the “frame problem” (Shanahan, 1997) in Artificial Intelligence. As a result, a direct approach to coding an *exact* set of fixed-point attractors (not allowing additional ones) produces a formula whose size is proportional to the number of states (i.e., exponential in the number of molecular species). Such an approach is not scalable. Moreover, if we are looking for solutions with no cyclic attractors, then coding this into a Boolean formula results in an intractable combinatorial explosion even for small networks.

Armed with Dubrova and Teslenko's (2011) method for detecting attractors in synchronous Boolean networks, *Griffin* does not block all unwanted attractors in the initial formula, but performs a “lazy” blocking, gradually adding the subformulas of unwanted attractors that appear in potential solutions. This technique, called “clause learning” (Franco and Martin, 2009), is used in SAT solvers, but at a lower level than *Griffin* does.

Table 2 shows how the gradual application of biological constraints at runtime is able to tackle all queries in our case studies. *Griffin* exhibits a significant increase in scalability as compared with the 2014 prototype of *Griffin* that did not have this feature.

**Table 2:** Scalability increase through clause learning in *Griffin*.

**R-graph size**				**No. of Boolean networks**	**Exclusion of attractors**		
***n***	***m***	**indegree**		**$\prod_{i=1}^{n}2^{\left(2^{d_{i}}\right)}$**	**No. of exclusionary clauses**	***Griffin***	

		***d***	#		$\sum_{k=1}^{2^{n}}\left(2^{n}\right)!/\left(k\left(2^{n}-k\right)!\right)$	****NE****	****CL****
**Small R-graphs with constant indegree**							
1	1	1	1	4	3	✓	✓
2	2	1	2	16	24	✓	✓
2	4	2	2	256			
3	3	1	3	64	16, 072	✓	✓
3	6	2	3	4,096			
3	9	3	3	16,777,216			
***A. thaliana* root (questions 5 and 6)**							
11	50	1	1	4.9 × 10^173^	2.2 × 10^5,891^	✗	✓
		3	2				
		4	3				
		5	2				
		6	1				
		7	1				
		8	1				
***A. thaliana*** **modified root (question 7)**							
11	90	4	1	4.8 × 10^1,661^	2.2 × 10^5,891^	✗	✓
		7	1				
		8	5				
		9	2				
		10	1				
		11	1				
***A. thaliana*** **flower (questions 1**, **2, and 3)**							
13	45	1	5	1.3 × 10^151^	4.2 × 10^28,499^	✗	✓
		2	1				
		3	1				
		4	2				
		6	2				
		7	1				
		8	1				
***A. thaliana*** **sepal (question 4)**							
21	32	1	12	2.9 × 10^20^	8.6 × 10^12,346,634^	✗	✓
		2	7				
		3	2				

*The first four columns (from the left) are the R-graph parameters used in a query. The first column shows the number of vertices, n; the second column shows the number of edges, m; the thrid column, labeled d, is an indegree, while the fourth column, labeled #, is the number of vertices in the R-graph having indegree d. The fifth column, no. of Boolean networks, is an upper bound of the number of satisfying Boolean networks. The last three columns give an idea of the difficulty of achieving an exclusion of attractors in Griffin's solutions through two different strategies. These columns show an estimate of the number of clauses required to exclude all possible attractors (column labeled no. of exclusionary clauses) and whether adding such constraint is tractable using naive encoding (column labeled NE) or using clause learning (column labeled CL). Naive encoding is the strategy used in early versions of Griffin and consists of adding in advance all constraints to the formula that prohibit the unwanted attractors. With naive encoding, the size of the formula required to enforce an exact set of desired attractors is too big in all case studies presented here (although question 4 do not ask for such condition), while clause learning allows Griffin to provide answers to all queries. Clause learning endows Griffin with a significant improvement in scalability*.

**Algorithm 2:**
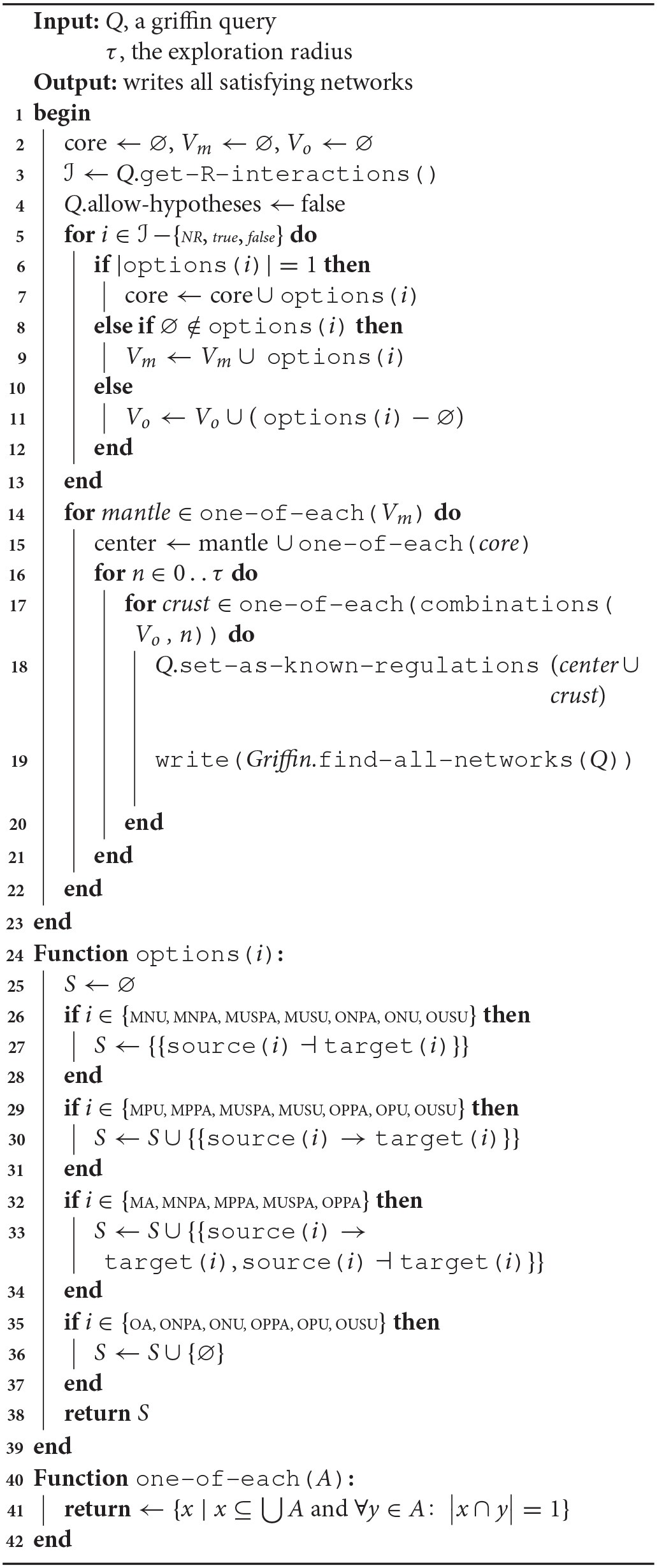
Radial exploration strategy for query splitting

#### 2.1.4. Partially known state transitions

*Griffin* is able to represent partially known state transitions, in particular those corresponding to fixed-point attractors where the value of some species is unknown.

#### 2.1.5. Whole queries and query splitting

**Figure 1:**
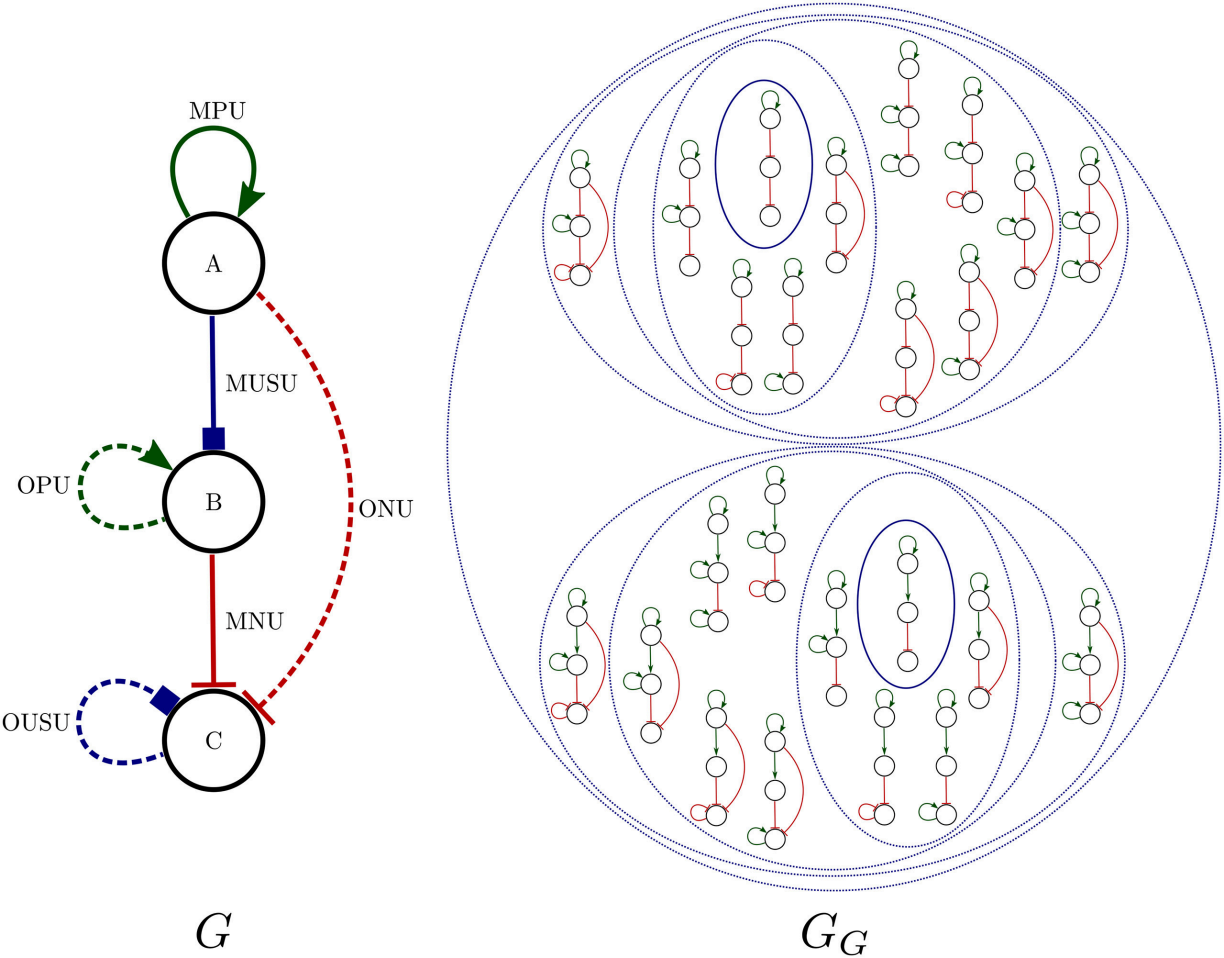
**Left:** An R-graph *G*. **Right:** The set of all instantiations of *G*, *G*_*G*_ (see definition 12). In the radial exploration strategy used by *Griffin* there are two centers contained in the continuous ovals. The nested dotted ovals illustrate regulation graphs that are at a certain radius from each center: continuous oval for radius 0, inner dotted ovals for radius 1, second inner dotted ovals for radius 2 and so on. In this example, each regulation graph of *G*_*G*_ corresponds to an R-graph of a query member of a query splitting. The strategy used for this query splitting is shown in Algorithm 2. In general, the number of queries in the query splitting does not correspond to the number of instantiations of an R-graph, due to the fact that a member of the splitting may not have satisfying solutions, while all instantiations of *G* have satisfying solutions.

It is also possible to have certain user control in the search for Boolean networks through an operating mode called *query splitting*. In the query-splitting mode, *Griffin* can (1) first instantiate an R-graph into all possible ordinary regulation graphs, (2) next obtain answers for each such instance, and (3) finally combine all resulting answers. It may be useful to partition a problem this way, as the time required to solve all instances might be less than the time required for solving the original query. Figure 1 shows an example of query splitting using the “radial” exploration strategy (see Algorithm 2).

**Algorithm 3:**
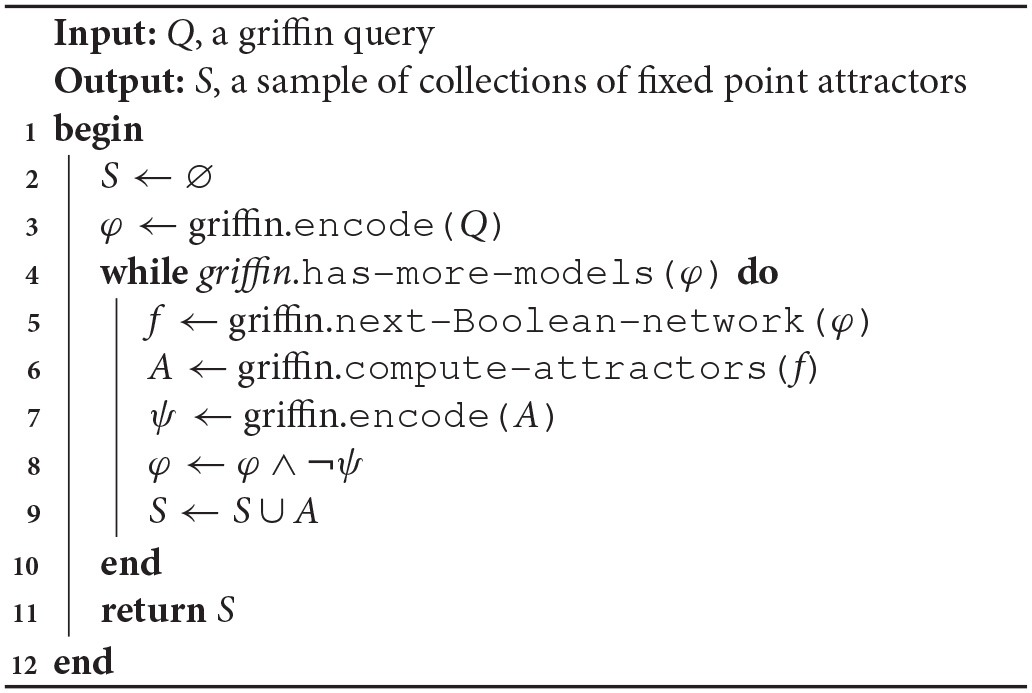
Exploration of sets of fixed point attractors

In the radial exploration strategy, an ordinary regulation graph resulting from removing all optional regulations of an R-graph, called a *center*, is obtained. Next, combinations of optional regulations are added to the obtained center. The number of optional regulations added to the center is called the *radius*.

#### 2.1.6. *Griffin*'s structure

**Figure 2:**
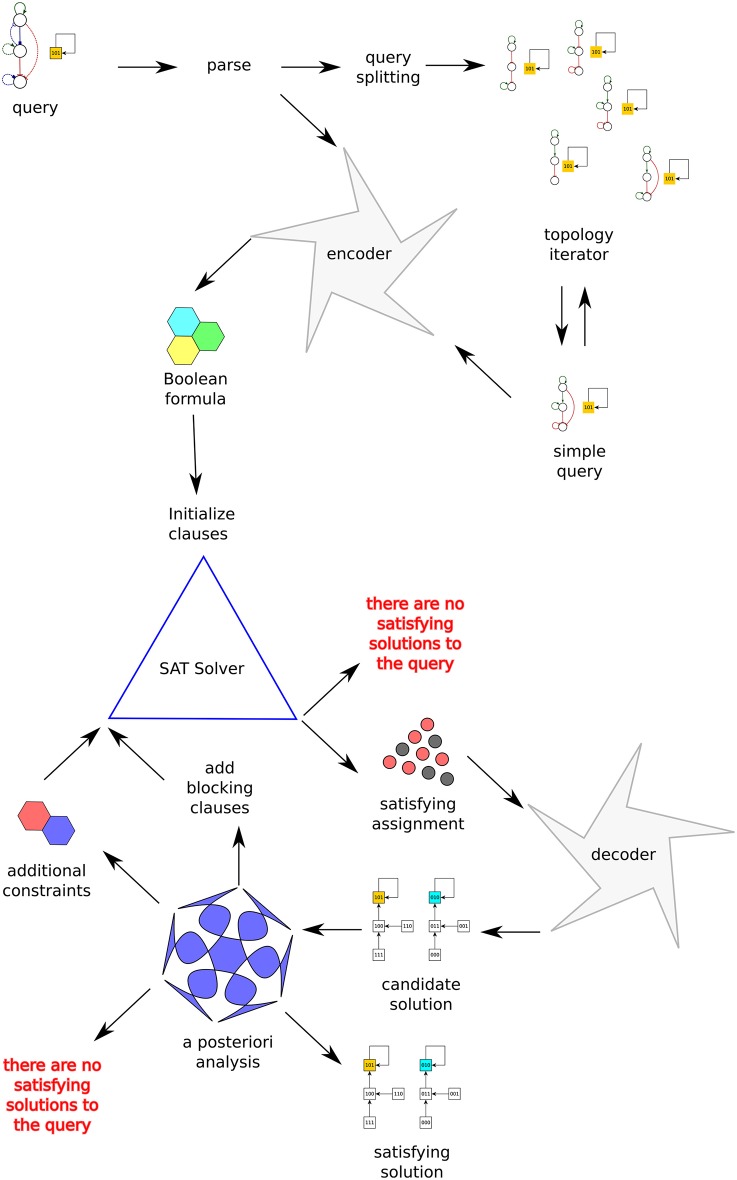
Schematic of *Griffin's* information flow. The query at the top-left corner is first parsed. Depending on the options it is either directly encoded into a Boolean formula or split into simpler queries. The Boolean formula is fed into a SAT solver. Each satisfying assignment returned by the SAT solver is decoded into a synchronous Boolean network, a candidate solution. It is also possible that the SAT solver concludes that the formula is a contradiction, that is, there are no synchronous Boolean networks satisfying the query. If a candidate solution is found, blocking constraints prevent finding the same solution twice. Further properties of the candidate solution are verified, possibly resulting in the generation of additional constraints. The *runtime blocking* analysis concludes with the rejection or acceptance of the candidate solution. If no candidate solution passes the *runtime blocking* analysis, then *Griffin* concludes there are no satisfying solutions to the query.

Algorithm 1 shows the exploration strategy used by *Griffin* to find the set of satisfying solutions to a query. Figure 2 depicts the processing of a query by *Griffin*.

### 2.2. Case studies

We demonstrate *Griffin*'s functionalities with three different Boolean network models taken from the literature. The first example allows us to illustrate queries for (a) finding Boolean networks given an R-graph, (b) finding the sets of fixed-point attractors of the Boolean networks of a given R-graph, and (c) finding Boolean networks given an R-graph and a desired set of fixed-point attractors.

The second example is devoted to hypothetical regulations. Such regulations will enable us to exemplify *Griffin*'s whole-query vs. query-splitting approaches.

The third example shows how uncertainty in the steady state behavior of a system can be effectively expressed by combining partially known fixed-point attractors and explicit exclusions of fixed-point attractors.

Some features, such as the use of constraints on mutants and specification of desired cyclic attractors, have been left out from this article. However, we have illustrated the use of these capabilities in previous articles (Rosenblueth et al., 2014; Weinstein et al., 2015; Azpeitia et al., 2017; García-Gómez et al., 2017).

#### 2.2.1. First case study: *Arabidopsis thaliana* flower model

We start by taking the *A. thaliana* floral organ determination gene regulatory network model developed by Alvarez-Buylla et al. (2010) (henceforth referred to as *A. thaliana* flower model). In their work (Mendoza and Alvarez-Buylla, 1998; Mendoza et al., 1999; Espinosa-Soto et al., 2004; Alvarez-Buylla et al., 2010), these authors report that this network is capable of reproducing the stable gene expression observed during the development of the flower organs, plus some inflorescence stages. Observe that these authors do not use a formal definition of regulation. Moreover, the search for networks satisfying the regulation graph (built from the literature) is done manually and without employing the expected set of fixed points to guide the search. Normally, these authors report only *one* Boolean network consistent with the regulation graph and recovering a set of fixed points coherent with the experimental information. We will show our attempts at reproducing their last results (Alvarez-Buylla et al., 2010) with *Griffin*.

##### 2.2.1.1. Regulation graph as input

We first illustrate a direct attempt at trying to recover the 10 known fixed-points (i.e., steady states) for the model of *A. thaliana* without using the desired set of fixed-points as input. This approach assumes that genes do not oscillate. This assumption can be justified by observing that even with such a constraint we did not recover the desired set of steady states. Hence, if we allowed for the possibility of cyclic attractors, thus enlarging the size of the search space, we would be even less likely to recover the desired set of fixed-point attractors.

**Figure 3:**
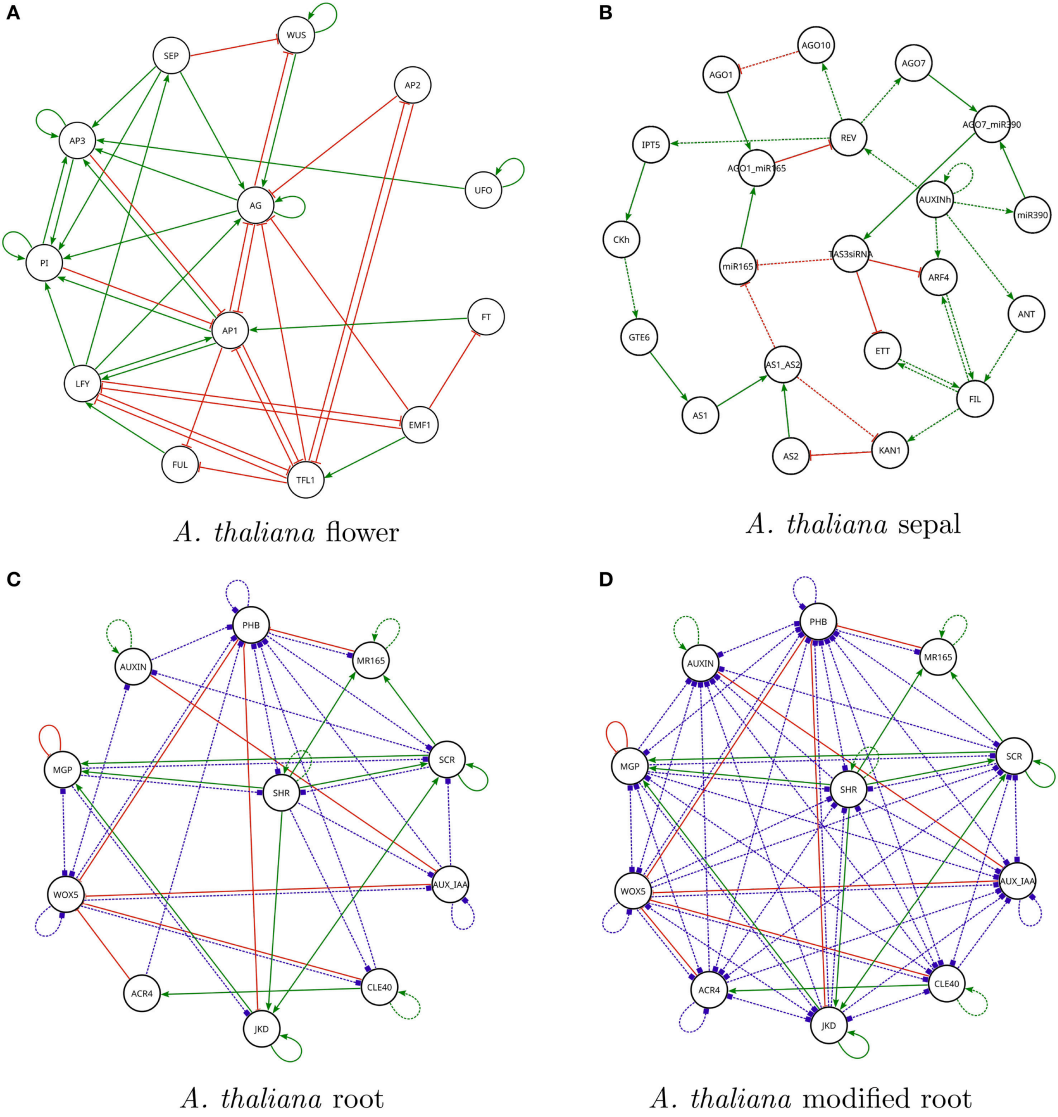
Regulation graphs for *A. thaliana* models used in the section Case Studies. **(A)** Flower development model of Alvarez-Buylla et al. (2010). Nodes *LUG* and *CLF* have been omitted from the diagram because they are constant inputs to the model. **(B)** Sepal primordium polarity model of La Rota et al. (2011). For all figures: positive regulations are shown as green lines with arrow tips, negative regulations are shown as red lines with orthogonal line tips, dashed lines represent hypothetical interactions (it is unknown whether they are present or not). Blue dashed lines with square tips represent hypothetical interactions of unknown sign. **(C)** Root cell stem niche model of Azpeitia et al. (2013). **(D)** Modified root cell stem niche model of Azpeitia et al. (2013) is shown with several new hypotheses.

Question 1. *The first question we asked Griffin was*: What are the *Boolean networks* satisfying the known regulation graph of the *A. thaliana* flower model (Figure 3A) and that do not have cyclic attractors?

Listing 1 in Supplementary Material shows the corresponding query as posed to *Griffin*. *Griffin*'s answer was positive, giving a large number of satisfying Boolean networks before exhausting the available resources. We hence limited to 100 the number of answers. These answers, however, were similar to each other, and none of them had the desired set of fixed-point attractors. Consequently, we devised search strategies to look for diversity in the answers.

Question 2. *What are the possible distinct* fixed-point attractor sets *(FPASs) for Boolean networks compatible with the known regulatory interactions of *A. thaliana* flower model and that do not have cyclic dynamics?*

Normally, *Griffin* can be used through a query language. However, if such a language is not expressive enough for the desired query, it is possible to run *Griffin* through its application programming interface (API). Algorithm 3 exemplifies the use of *Griffin* via its API. This algorithm computes distinct FPASs. To prevent the possibility of finding the same combination more than once, there is a blocking constraint representing the negation of that particular FPAS.

It is important to note that this search algorithm is not complete, in the sense that some FPASs may be excluded from the answer as a result of the blocking strategy. Adding a blocking clause not only prevents a particular FPAS from being found again; it also blocks all FPASs that are a superset of the FPAS of a satisfying solution found by *Griffin*. If the whole set of FPASs is required, this algorithm would have to be modified with more sophisticated blocking techniques. In our case, this was not deemed necessary because the computation of the sample of FPASs already exhausted the computational resources.

**Figure 4:**
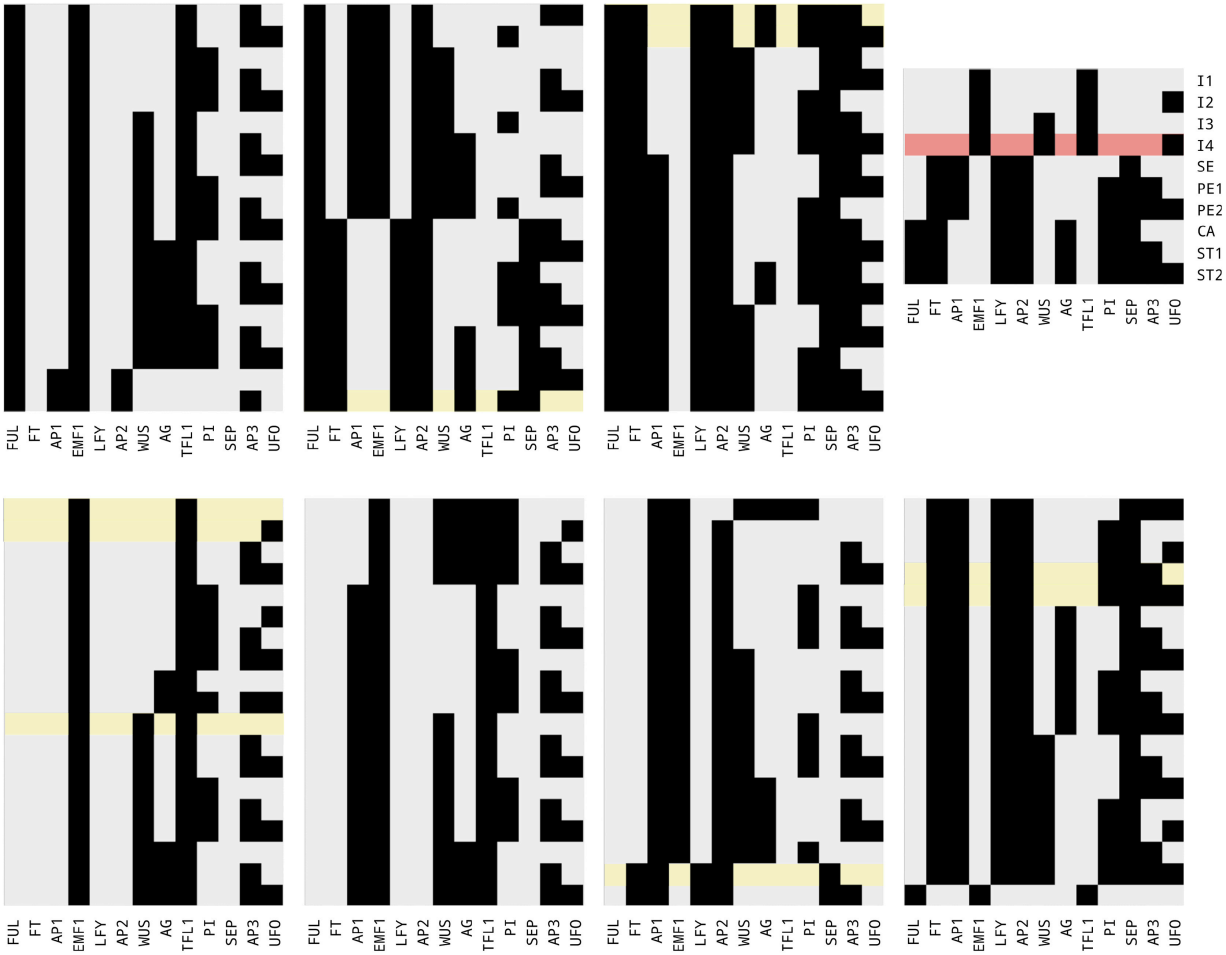
A sample of 133 fixed-point attractors that are compatible with the interaction graph of Figure 3A. Black squares represent expressed genes; gray squares represent unexpressed genes. From bottom-left to top-right, there are seven groups of 19 rows and 13 columns each. Each column is labeled with a particular gene. Each row is an expression profile for the 13 genes and corresponds to a compatible fixed-point attractor for the regulations present in the interaction graph. At the top-right corner the 10 known fixed-point attractors corresponding to the cell types: inflorescence shoot apical meristem *I1, I2, I3, I4*; sepal *SE*; petal *PE1, PE2*; stamen *ST1, ST2*; and carpel *CA*. Nine of the 10 fixed-point attractors present in the sample have been highlighted in green. The algorithm used to generate the sample did not find the *I4* profile highlighted in red.

*Griffin* found 2,896 Boolean networks. The sets of attractors were not only distinct but some of them also had distinct cardinalities. The minimum number of fixed-point attractors for the networks in the sample was two; the maximum was 15. We found 133 different fixed-point attractors in such a sample. Of the 10 desired fixed-point attractors that are compatible with the regulation graph for *A. thaliana* flower (Figure 3A), *Griffin* was only able to find nine, as explained in Figure 4.

**Figure 5:**
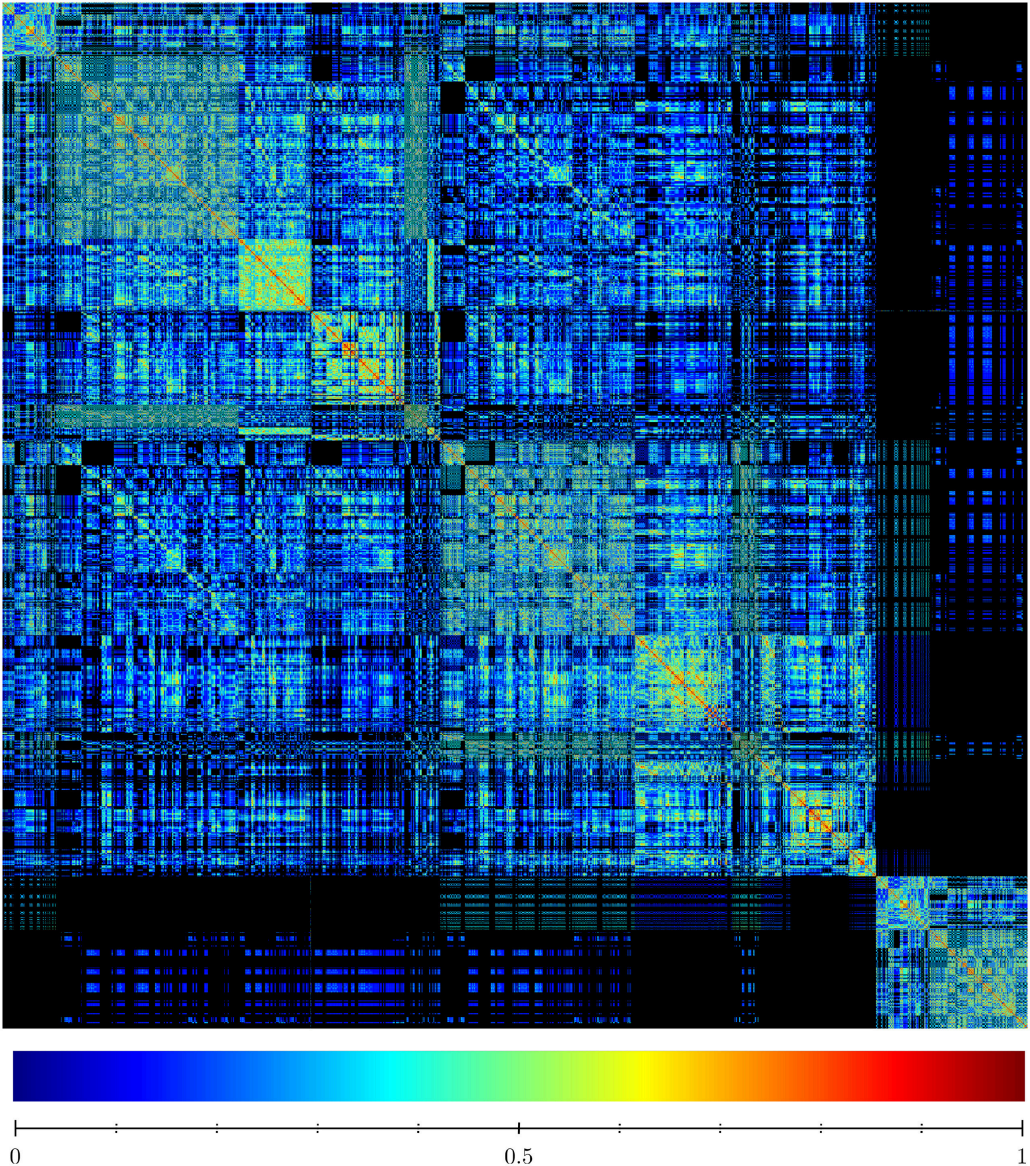
Similarity matrix of sets of fixed-point attractors for a sample of 2,896 Boolean networks satisfying the interaction graph of *A. thaliana* flower model shown in Figure 3A and that do not have cyclic attractors. The similarity matrix shows the degree to which different sets of fixed-point attractors share the same elements. Each row and column represents a particular witness. The entry (*i, j*) represents the similarity between the fixed-point attractor sets of witness *i* and witness *j*. The maximum similarity between two networks equals 1 if they have the same set of fixed-points. Algorithm 3 ensures that the witnesses have different sets of fixed-point attractors. Therefore, the only entries in the similarity matrix that are equal to one are in the diagonal. The minimum similarity between two networks is zero. This happens when the intersection of their sets of fixed-point attractors is empty. High values of similarity are colored red while low values of similarity are colored blue. Zero similarity corresponds to black. The similarity of two sets was found using the cosine similarity given by $sim(x,y)=\frac{x\cdot y}{\left\|x\right\|\left\|y\right\|}$ where *x* and *y* are binary vectors encoding the presence or absence of different fixed-point attractors.

**Figure 6:**
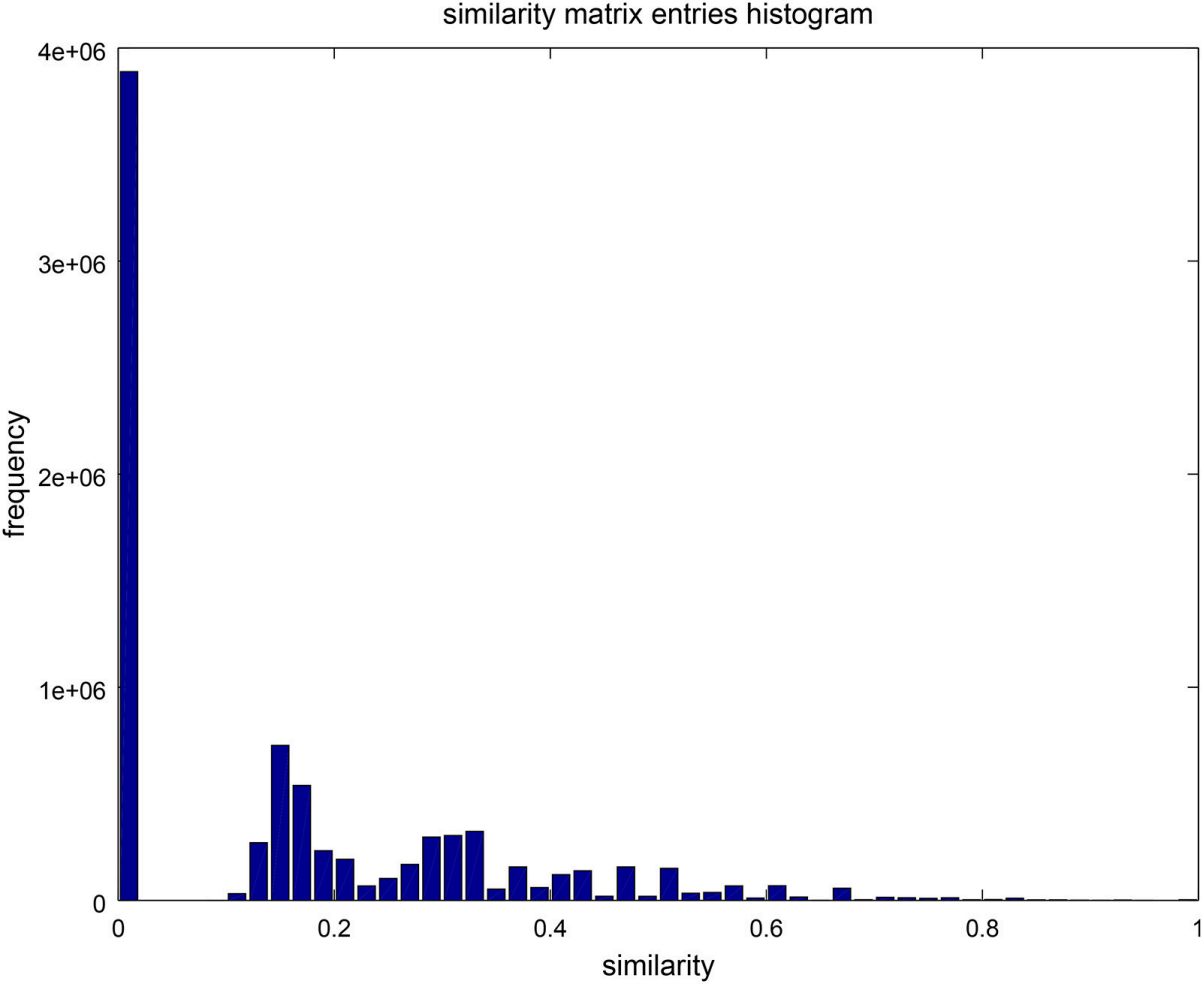
A histogram with 30 bins, showing the distribution of entries in the similarity matrix of Figure 5. Low similarity values are more frequent than high similarities reflecting high diversity in the sets of fixed-point attractors.

To assess the diversity of the sample, we analyzed the similarity of the FPASs between each pair of found networks. Figure 5 is a visualization of the similarity matrix computed for the 2,896 networks in the sample. The visualization shows not only that many FPASs share common elements, but also that there are significant number of them that do not share any fixed-point attractor (2,896^2^ × 0.46 entries in the matrix are zeros; that is, 46% of pairs are completely different in their fixed-point attractors). Our conclusion is that results produced by *Griffin* show that there is a significant diversity of fixed-point attractors in Boolean networks with dynamics compatible with a given regulation graph (see Figure 6).

Note that a regulation graph (without biological constraints) can be satisfied by a vast number of Boolean networks corresponding to different dynamic behaviors. Therefore, in addition to the regulation graph, biological constraints on *Griffin*'s input (as the set of desired attractors) are important for producing more accurate Boolean networks.

We show next that *Griffin* was able to find fully compliant Boolean networks once steady-state constraints were added to the query.

##### 2.2.1.2. Regulation graph plus expected set of attractors as input

Question 3. *The third question we asked Griffin was*: Are there any Boolean networks satisfying the known regulatory interactions of the *A. thaliana* flower model that have exactly the known set of fixed-point attractors?

Listing 2 in Supplementary Material has a new constraint with respect to the previous query, asking *Griffin* to limit the search to those Boolean networks having exactly the set of known fixed-point attractors of Figure 4. As with the previous question, *Griffin*'s answer was positive, giving a large number of satisfying Boolean networks. Before exhausting the resources, *Griffin* found 328,565 Boolean networks satisfying the constraints. We observe that were it not for the fact that *Griffin* blocks attractors *on the fly* (as opposed to blocking unwanted attractors in advance), the resources would be exhausted when building the formula (given the length of the formula required for blocking any additional fixed-point or cyclic attractor). After adding to the input the set of desired attractors, the number of solutions was still vast. *Griffin* allows adding constrains that could reduce even more the number of solutions. An example of such constraints is the effect of mutations on the set of attractors (Rosenblueth et al., 2014; Azpeitia et al., 2017).

**Figure 7:**
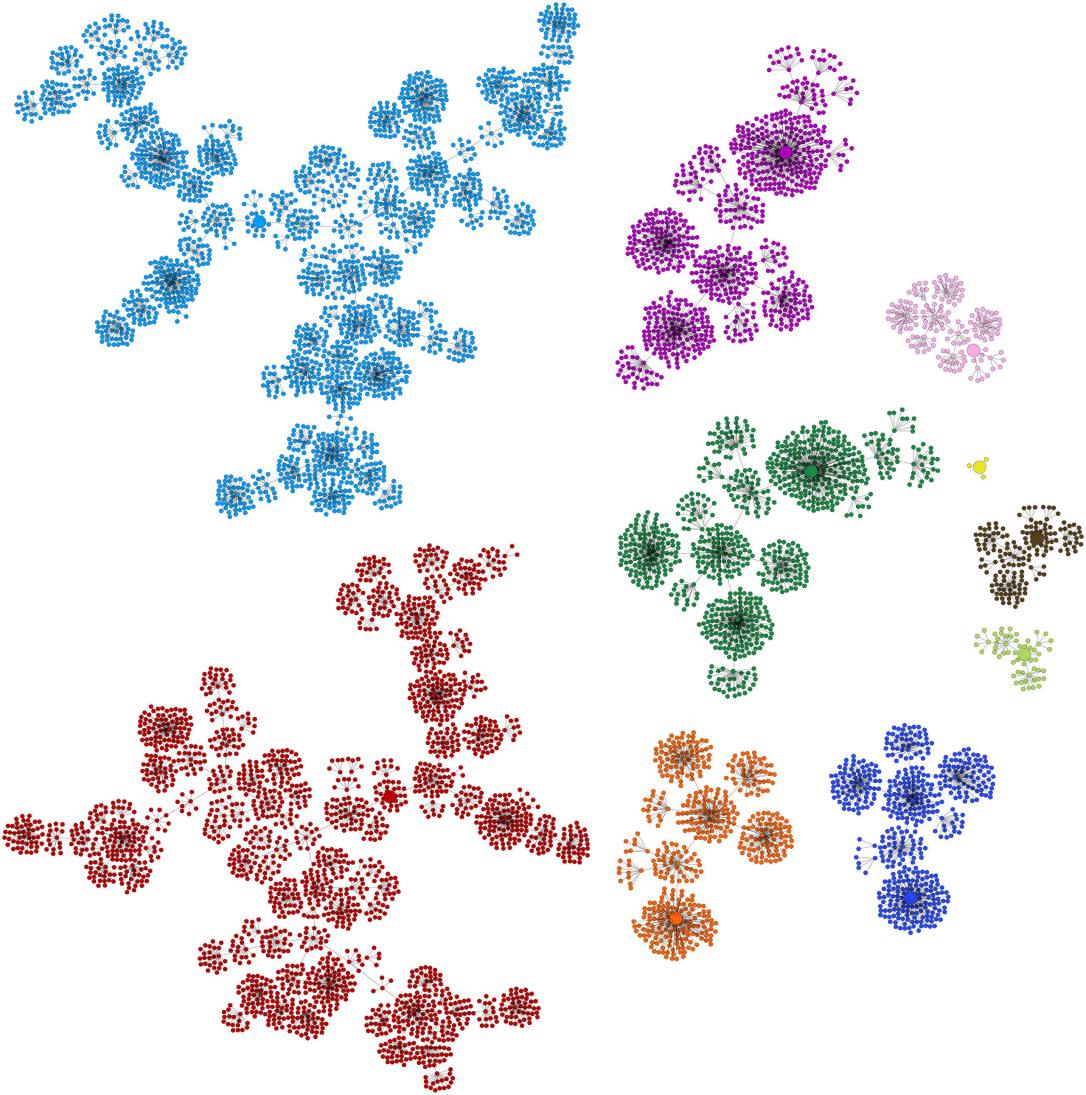
Depiction of basins of attraction for the ten known fixed-point attractors corresponding to the Boolean network having the combination of basin sizes reported in the sixth row in Table 3. The biggest component with 2,488 states corresponding to ST2 is colored red, the second biggest with 2,432 states, corresponds to CA and is colored light blue, I1 is colored purple, I2 dark green, I3 dark blue, I4 orange, SE brown, PE1, the smallest component with only four states, is colored yellow, PE2 pink and ST1 light green. The fixed-point attractors are represented by bigger circles than those of non stationary states. *Griffin* use the algorithm of Dubrova and Teslenko (2011) to compute the attractors of the Boolean networks. Binary Decision Diagrams and the backward reachable set algorithm of Garg et al. (2008) are applied to each attractor to compute their corresponding basins.

As well as inferring Boolean networks, *Griffin* can be used to perform analysis, as we show next. Figure 7 depicts a state space diagram for the 10 basins of attraction for the network having the combination reported in the sixth row of Table 3. Our results would suggest that gene regulatory networks are robust allowing them to be stable in developmental and evolutionary scales, as observed for flowers (Drinnan et al., 1994).

**Table 3:** Frequency of sizes of basins of attraction for the 10 known fixed-point attractors of Figure 4 in a sample of 328,565 Boolean networks found by *Griffin* that recover the exact set of fixed-point attractors.

**I1**	**I2**	**I3**	**I4**	***SE***	**PE1**	**PE2**	**CA**	**ST1**	**ST2**	**Num. of networks**
896	896	512	512	2,596	4	2,592	48	40	96	156,227
884	884	524	524	2,596	4	2,592	48	40	96	95,352
908	908	500	500	2,596	4	2,592	48	40	96	57,725
968	968	440	440	2,596	4	2,592	48	40	96	19,259
896	896	512	512	2,588	4	2,584	52	44	104	1
916	916	524	524	164	4	168	2,432	56	2,488	1

*Cyclic attractors were prohibited in the query. There are only six different combinations of basin sizes. The last column shows the number of Boolean networks having the corresponding combinations of basin sizes*.

#### 2.2.2. Second case study: *A. thaliana* sepal primordium polarity

La Rota et al. (2011) developed a model of sepal primordium polarity in the young flower of *A. thaliana*. These authors defined a set of expected attractors by analyzing the expression patterns of the most important genes during sepal development. Then, using published data, they defined the set of known regulatory interactions and looked for putative binding sites indicating putative missing regulatory interactions. Finally, using mathematical programming, they built Boolean networks that conformed with the known and missing interactions to verify if any of such networks could produce the set of expected attractors. We chose this example as it enables us to illustrate how incomplete knowledge about the regulators of specific genes can be captured in a *Griffin* query. In the following subsection, we also illustrate how to set a query-splitting strategy.

##### 2.2.2.1. Hypothetical regulations

The model of sepal primordium polarity for *A. thaliana* of La Rota et al. (2011) (henceforth referred to as *A. thaliana* sepal) was integrated considering incomplete knowledge and uncertainty in the data. After analyzing the data, these workers concluded that the model based on experimental data was unable to obtain the set of expected attractors. Then, based on bioinformatic data, they included a number of hypothetical missing regulations that could be necessary to obtain the desired attractors. The computational methods used by La Rota et al. (2011) were based on mixed integer linear programming representations and iterative procedures. They used their algorithm to reproduce steady states and regulatory interactions to be functional, as well as other biologically meaningful constraints. We were interested in testing *Griffin*'s ability to find satisfying solutions to this problem.

Question 4. *The question we asked Griffin was*: Are there any Boolean networks satisfying the known and hypothetical regulations of *A. thaliana* sepal model that have the known set of fixed-point attractors with no constraints on possible additional attractors?

Listing 3 in Supplementary Material shows the *Griffin* query used to answer the previous question. It is simple to list hypothetical regulations, such as the 19 hypotheses formulated by La Rota et al. (2011) in their regulation graph (Figure 3B [based on Figure 3D of La Rota et al., 2011]). By construction, the regulations of satisfying networks found by *Griffin*, if any, are functional (Rosenblueth et al., 2014). Therefore, no special instruction is needed to specify this requirement. We included the expected fixed-point attractors as a constraint. Additionally, we asked *Griffin* to perform query splitting (see definition 16, below) on the question using the radial exploration strategy of Algorithm 2. By varying the query option corresponding to the radius, *Griffin* limited the number of simultaneous hypotheses considered at a time for each query *q* ∈ *s*(*P*) in the splitting (see definition 16).

**Figure 8:**
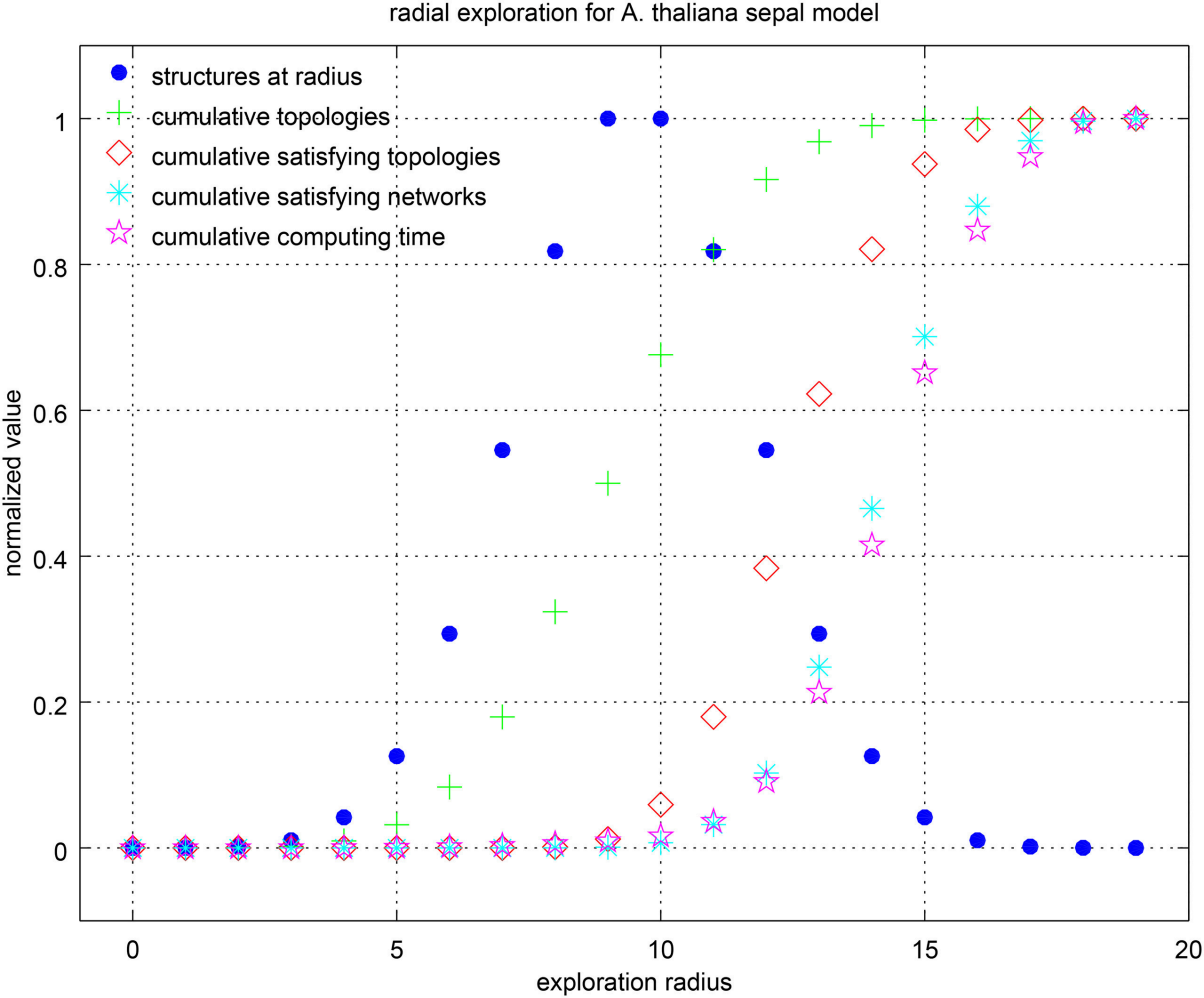
Center-radius exploration strategy for the sepal model. Values have been normalized by their maximum in each category. The number of R-graphs grows rapidly with the radius. It is given by the binomial coefficient $\left(\begin{array}{c} h\\ r \end{array}\right)$, where *h* is the number of hypotheses and *r* the radius. It can be seen that the computing time is highly correlated with the number of cumulative satisfying networks.

We asked several queries varying the radius (paragraph “paragraph:query-splitting”). As there are 19 hypotheses, this is also the maximum possible radius. Table 4 summarizes the results. It can be seen that having control on the radius provided valuable information about the hypotheses. In particular, *Griffin* found all combinations of hypothetical regulations that reproduced the expected attractors, including the smallest set of required hypothetical regulations to obtain the attractors. In this case, there were no satisfying networks below a radius eight. In other words, the simplest models able to satisfy the constraints must include the right combination of at least eight hypotheses. There were 32 solutions at this radius, and only eight different right combinations (same number of associated interaction graphs). *Griffin* was able to compute all the solutions for the query, a total of 439,296. Notice that in the R-graph of this example, there are 19 hypothetical regulations, which is equivalent to 2^19^ = 524, 288 ordinary regulation graphs. *Griffin* analyzed all these possible ordinary regulation graphs in a single question. We can also see the difference between the cardinality of the query splitting |*s*(*P*)| = 524, 288 and the cardinality of the instantiations of the corresponding R-graph |*G*_*G*_| = 6, 912. That is, only about 1.32% of the ordinary regulation graphs produced solutions. To measure the computational cost of the query-splitting strategy, we compared the computing time for a single query (with no query-splitting strategy). This example shows that making 524,288 questions needed fewer computational resources than a single complex question did. Figure 8 shows graphically the information of the table. It can be seen that the computing time correlates well with the number of solutions found.

**Table 4:** Satisfying R-graphs and Boolean networks found by *Griffin* for the center-radius exploration strategy applied to *A. thaliana* sepal model.

**Radius**	**Regulation graphs at radius**	**Cumulative regulation graphs**	**Cumulative satisfying Regulation graphs**	**Cumulative satisfying networks**	**Cumulative computing time [h:m]**
8	75,582	1,69,766	8	32	0:01
9	92,378	2,62,144	84	448	0:03
10	92,378	3,54,522	410	3,120	0:05
11	75,582	4,30,104	1,243	14,048	0:11
12	50,388	4,80,492	2,651	45,120	0:28
13	27,132	5,07,624	4,303	1,08,912	1:06
14	11,628	5,19,252	5,675	2,04,496	2:08
15	3,876	5,23,128	6,481	3,07,968	3:22
16	969	5,24,097	6,809	3,86,496	4:22
17	171	5,24,268	6,897	4,25,968	4:54
18	19	5,24,287	6,911	4,37,728	5:08
19	1	5,24,288	6,912	4,39,296	5:10

*Each row summarizes the results of individual runs. The first column shows the radius. The second column has the corresponding binomial coefficient giving the number of combinations of active hypotheses at each radius. The third column has the cumulative number of R-graphs. Not all R-graphs are consistent with the specified constraints. As we can see in the fourth column that gives the total number of satisfying R-graphs for each run, it is necessary to add at least eight simultaneous hypotheses to find satisfying networks. Each satisfying R-graph can have one or more satisfying Boolean networks. The total number of satisfying networks is shown in the fifth column. The processing time for each query is shown in the last column. As a comparison of performance, we also timed the query without using the center-radius strategy. The elapsed time was of 5:14 h. This illustrates that, in this case, splitting the query into several simpler ones (the third column gives the exact number of queries run), does not add time. The center-radius strategy finished over 4 min before the single query did*.

#### 2.2.3. Third case study: *A. thaliana* root stem cell niche

The *A. thaliana* root stem cell niche is a model system for the study of plant development and stem cell niche dynamics. We consider here the work of Azpeitia et al. (2013). These authors have used multiple strategies for finding missing gene interactions able to explain the observed experimental steady state dynamics. Their study faces the computational complexity of searching within the space of potential Boolean functions, which is large and grows exponentially in the number of regulator genes. Here, we review some of their proposed procedures for predicting missing interactions, expressing such procedures as symbolic constraints. In fact, we have previously used *Griffin*(Rosenblueth et al., 2014) to reproduce and extend (by finding other satisfying Boolean networks) a previously published Boolean network for *A. thaliana* root stem cell niche (Azpeitia et al., 2010). In this subsection, we illustrate how *Griffin* can deal with uncertainty in the gene interactions as well as in the attractors.

##### 2.2.3.1. Using R-graphs to express hypotheses

In the next question, we explore the root stem cell niche model of Azpeitia et al. (2013) (henceforth referred to as *A. thaliana* root model). From the point of view of the Boolean network inference problem, this model is interesting because it is rich in hypotheses, that is, it incorporates uncertainties about the existence or sign of its regulatory interactions.

Question 5. Does there exist a Boolean network satisfying the *A. thaliana* root model?

The *A. thaliana* root model includes 30 hypothetical regulations as well as 20 known regulations. Figure 3C shows the set of regulations and Listing 4 in Supplementary Material exhibits the query file for the corresponding question. *Griffin*'s answer to the query was negative. This is compatible with the results reported by Azpeitia et al. (2013). *Griffin*'s conclusion, however, is important for the following reasons:

1. The search space of Boolean functions is vast. The number of regulators for each gene is as follows (see Figure 3C): *ACR4*, 1; *AUX_IAA*, 4; *AUXIN*, 3; *CLE40*, 3; *JKD*, 5; *MGP*, 5; *MR165*, 4; *PHB*, 8; *SCR*, 6; *SHR*, 4; *WOX5*, 7. The total number of possible Boolean networks is then given by: 2^2^1^^ × 2^2^4^^ × 2^2^3^^ × … × 2^2^7^^ ≈ 4.9 × 10^174^.
2. Azpeitia et al. (2013) performed extensive exploration of the search space, applying a set of procedures designed to reduce the complexity of the search and to focus on solutions with biological relevance. Their technique incorporated a heuristic search that tested combinations of hypothetical regulations adding each regulation one at a time. After three months of testing no solution was found. Because only a portion of the search space was explored, it was not possible to conclude whether or not there were satisfying Boolean networks.
3. When *Griffin*'s answer is negative, i.e., when it concludes that the query is unsatisfiable, its conclusion is often found as a consequence of a trivial contradiction in the Boolean formula given to the SAT solver. If that is the case, the answer is found immediately. In our case, however, *Griffin* took 6.8 ms to give the answer.
4. Despite the size of the search space, this example illustrates that *Griffin*'s *runtime* blocking strategy is effective. Inspecting *Griffin*'s log files, we learned that there is no trivial contradiction in the query formula. *Griffin* iterates over 22 candidate Boolean networks, rejecting all because they exhibit additional attractors. *Griffin* blocks 15 fixed-point attractors, five cyclic attractors of length two, and two cyclic attractors of length three. After blocking one of the incompatible attractors of the 22nd network, a contradiction is generated, allowing *Griffin* to conclude the nonexistence of a satisfying solution.

The fact that no network satisfied the constraints suggested that such constraints might be incorrect. After analyzing the assumptions of the model, we learned that there existed uncertainty about the specification of the fixed-point attractors of the model. In particular, the model contains (1) an hormone, auxin, whose distribution is graded along the *A. thaliana* root, and (2) a mobile peptide, CLE40, that diffuses from its expression domain. Neither the exact region where auxin is active nor the diffusion coefficient of CLE40 are known. Thus, their Boolean values in the attractors is not clear. *Griffin* is able to express such degree of uncertainty by the use of partially known fixed-point attractors. This is explored in the following subsection.

##### 2.2.3.2. Partially known fixed-point attractors

Uncertainty in stationary gene expression profiles when inferring the dynamics of a molecular network from the literature occurs frequently. To an extent, *Griffin* can deal with uncertainty by using partially known fixed-point attractors. We show in the following question that partially known fixed-point attractors, combined with explicit exclusion of certain fixed-point attractors, can be used to formulate complex hypotheses.

Question 6. Does there exist a Boolean network satisfying the *A. thaliana* root model having uncertainty in the definition of its steady-state behavior?

Listing 5 in Supplementary Material shows a modified query of the previous question. The constraints on the fixed-point attractors have been changed. Instead of just asking for nine well-defined fixed-point attractors, some uncertainty in the specification of them has been introduced by including partially known fixed-point attractors as well as prohibitions. A detailed explanation on the syntax and meaning of the constraint can be found in Table S1 in Supplementary Material. *Griffin* was able to find a satisfying solution after 2.9 s. This example shows that being able to express uncertainty (due to lack of information) in the attractors could also be important when inferring a molecular network, and that *Griffin* can easily express such uncertainty.

In the following example, we would like to significantly extend the number of hypothetical regulations of the *A. thaliana* root model (henceforth referred to as the modified *A. thaliana* root model), and test whether *Griffin* is able to cope with the complexity of the query.

Question 7. What are the Boolean network satisfying the modified *A. thaliana* root model having uncertainty in the definition of its steady-state behavior?

The modified *A. thaliana* root model included the 20 known interactions of the original model, as well as 70 hypothetical interactions, 66 of which were of unknown sign and four were positive. The corresponding R-regulation graph is shown in Figure 3D.

The significance of this example is twofold:

1. The search space of Boolean functions is significantly larger than that of the original *A. thaliana* root model. The number of regulators for each gene is as follows (see Figure 3D): *ACR4*, 8; *AUX_IAA*, 10; *AUXIN*, 9; *CLE40*, 7; *JKD*, 8; *MGP*, 8; *MR165*, 4; *PHB*, 11; *SCR*, 9; *SHR*, 8; *WOX5*, 8. The total number of possible Boolean networks is then given by: 2^2^8^^ × 2^2^10^^ × 2^2^9^^ × … × 2^2^8^^ ≈ 4.8 × 10^1, 661^.
2. *Griffin* required 5,520 variables to represent the update function in this problem, plus 160 regulation variables (the *R*^+^s and *R*^−^s) plus an unidentified number of switching variables required to represent the formula in an “equisatisfiable” conjunctive normal form (Tseitin, 1968).

**Figure 9:**
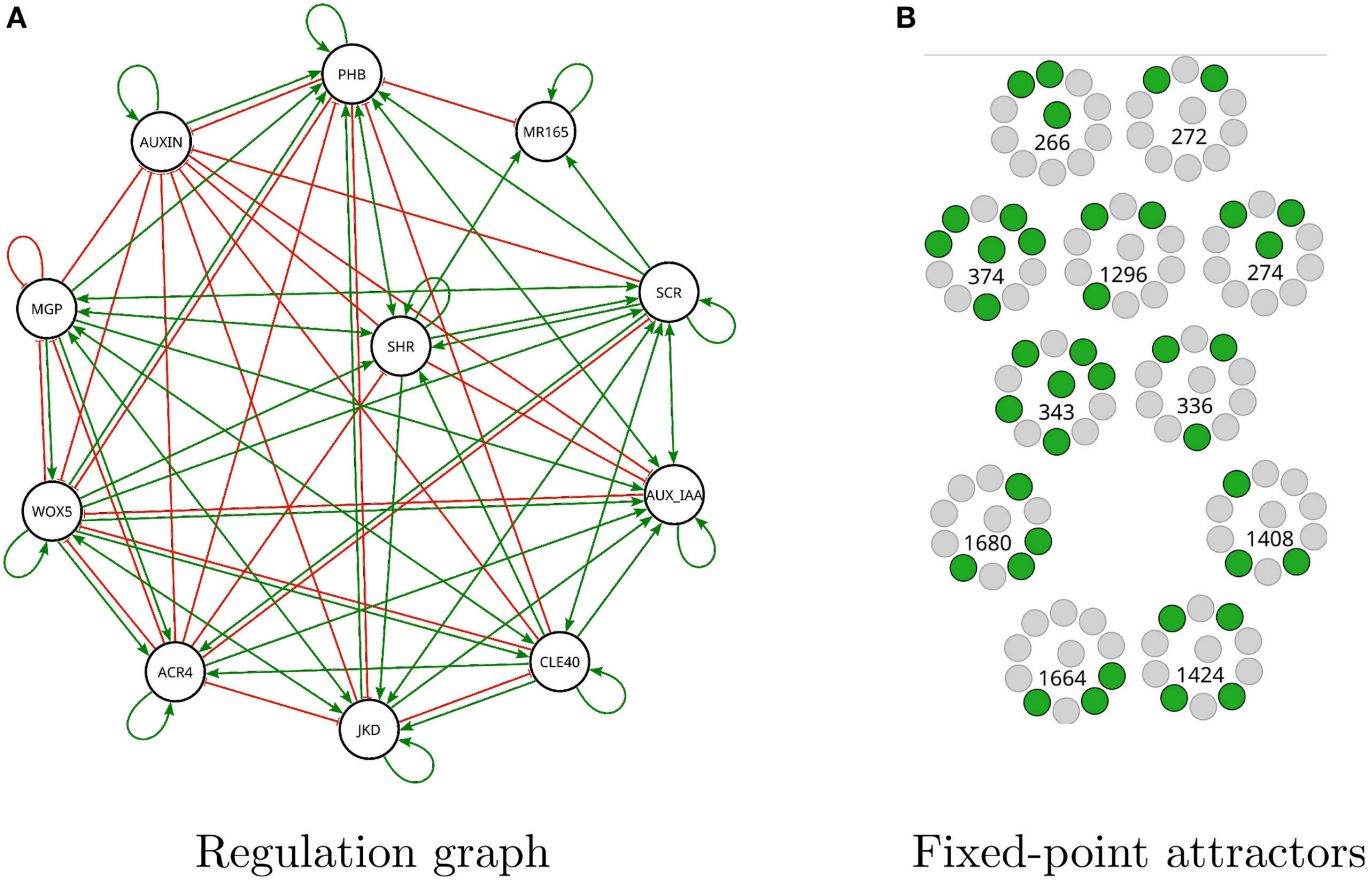
Modified *A. thaliana* root Boolean network found by *Griffin* satisfying question 7. **(A)** Interaction graph of the solution has 83 regulations of the 90 possible (see Figure 3D). **(B)** There are 11 fixed-point attractors in the solution. For each expression profile active genes are shown in green while inactive genes are shown in gray. The number corresponds to the decimal notation used in Table S1 in Supplementary Material.

The query for question 7 is shown in Listing 6 in Supplementary Material. A satisfying Boolean network was found after 24 min., which has a total of 83 regulations, as can be seen in Figure 9.

For questions 1, 2, 3, and 7 we ran *Griffin* on a Dell PowerEdge T320 Server with 80 GB of RAM. For the rest of the questions we used a laptop computer with Intel(R) Core(TM) i7-4710HQ CPU @ 2.50GHz and 16 GB of RAM.

## 3. Discussion

In this section, we first give a summary of *Griffin*. Next, we review methods for Boolean-network inference and related formalisms, so as to place *Griffin* in context within this area of research. Finally, we discuss possible directions for future work.

### 3.1. Overview of *Griffin*

In the inference of Boolean networks form regulation graphs, the fact that multiple networks might be consistent with the same (given) regulation graph is sometimes neglected. Often, publications reporting the inference of Boolean networks consider a single network. Even if several such networks are analyzed, only a fragment of the possibly vast number of consistent networks is usually considered. *Griffin*, by contrast, employs a formal definition of regulation (Naldi et al., 2007; Richard et al., 2012; Mori and Mochizuki, 2017) to build an implicit representation of all possible networks satisfying the regulation graph. *Griffin* then augments such a representation with biological constraints, resulting in a uniform representation of all potential networks and at the same time reducing such potential number of solution networks. This representation is then given to a powerful, symbolic search mechanism.

A key contribution to *Model Checking* (Clarke et al., 1999) was the incorporation of symbolic representations of data (Burch et al., 1992). Such representations allowed Model Checking to verify systems, first with 10^20^ states, and subsequently with many more. As with model checkers, *Griffin* is able to handle large systems because of also using symbolic data structures. There exist two main symbolic techniques: *binary-decision diagrams* (BDDs) and *SAT solvers*. *Griffin* employs both, for solving different subproblems. *Griffin* finds cyclic attractors with a SAT solver (Dubrova and Teslenko, 2011), whereas it computes basins of attraction (Garg et al., 2008) with BDDs. The top level in *Griffin*, which builds a Boolean formula representing all biological information and constraints, also employs a SAT solver. *Griffin* thus capitalizes on the phenomenal recent developments of such solvers. This dramatic progress has “resulted in speed-ups of many orders of magnitude that have turned many problems that were considered intractable in the 1980s into trivially solved problems now.” (Franco and Martin, 2009).

Another strong point of our tool is the concept of an R-graph, a generalization of ordinary regulation graphs. R-graphs represent advantages from both the user and the computational viewpoints. The user has available the whole repertoire of combinations involving the sign of a regulation and whether a regulation is compulsory or simply hypothetical. For example, the user can specify a hypothetical regulation that, if present, should be negative and cannot be positive. From a computational point of view, an R-graph encompasses all hypothetical regulations in one formula, thus avoiding having to perform many separate tests. Computer tools employing ordinary regulation graphs, by contrast, usually test all possible combinations of presence and absence of all hypothetical regulations, resulting in numerous analyses.

*Griffin* is hence not only an innovative but also powerful computer tool for the inference of Boolean networks. It is possible, for example, to detect erroneous inferences due to the experimental, error-prone nature of biological data. Regulations that were believed to be positive might in fact turn out to be negative or vice versa. *Griffin* has detected these situations, as reported in Azpeitia et al. (2017).

### 3.2. Related work

As a first comparison between our work and related articles, it is important to point out a difference in the type of input data. In this work, *Griffin* input is composed of (partial) information about the network topology (R-graphs) along with other data representing biological constraints. R-graphs contain information on genetic connectivity that was inferred from data obtained by direct measurement of gene expression or protein interaction. By contrast, inference methods proposed by other authors (e.g., Laubenbacher and Stigler, 2004) have an input composed of time series together with other data that capture information on the network dynamics. Time series, unlike the R-graphs, represent experimental data obtained by direct measurement of gene expression or protein interaction.

There are a number of tutorials on Boolean-network inference (D'haeseleer et al., 2000; Markowetz and Spang, 2007; Karlebach and Shamir, 2008; Hecker et al., 2009; Hickman and Hodgman, 2009; Berestovsky and Nakhleh, 2013). From these tutorials we can classify algorithms according to (1) the expected input, (2) the kind of model inferred, and (3) the search strategy.

Much of the effort in Boolean-network inference has been aimed at having a binarized time-series data as input. Hence, multiple methods have been proposed and some of them have been compared with each other (Berestovsky and Nakhleh, 2013). An influential method in this category is reveal (Liang et al., 1998) (employing Shannon's mutual information between all pairs of molecules to extract an influence graph; the truth tables of the update functions for each molecule are simply taken from the time series). The use of mutual information and time-series for the inference of Boolean networks continues to develop (Barman and Kwon, 2017), as well as alternative methods based on time series. Example are Han et al. (2014) (using a Bayesian approximation), Shmulevich et al. (2003), Lähdesmäki et al. (2003), Akutsu et al. (1999), Laubenbacher and Stigler (2004), and Layek et al. (2011) (using a generate-and-test method, generating all possible update functions for one gene and testing with the input data). Extra information can be included in addition to time-series data. For example, an expected set of stable states (Layek et al., 2011), previously known regulations (Haider and Pal, 2012) and gene expression data (Chueh and Lu, 2012) are used as an aid to curtail the number of possible solutions.

*Griffin* belongs to a second family of methods taking as input a possibly partial regulation graph (perhaps obtained form the literature). There are also approaches employing both time-series data and a regulation graph, such as Ostrowski et al. (2016).

A third important area of research is the development of algorithms taking as input temporal-logic specifications, based on Model Checking (Clarke et al., 1999). Works following this approach are: Calzone et al. (2006b), Mateus et al. (2007), and Streck and Siebert (2015).

As for the kind of model inferred, here we would be concerned with Boolean networks and similar formalisms. Among the nonprobabilistic approaches, we find synchronous Boolean networks, asynchronous networks based on Thomas's formalism (Bernot et al., 2004; Khalis et al., 2009; Corblin et al., 2012; Richard et al., 2012), and polynomial dynamical systems (Laubenbacher and Stigler, 2004). Typically, methods based on temporal logic infer Kripke structures, which are closely related to Boolean networks. Probabilistic models, one the other hand, include Bayesian networks, and have the advantage of being able to deal with noise and uncertainty.

From the search-strategy point of view, Boolean-network inference methods may employ a simple random-value assignment (Pal et al., 2005), exhaustive search (Akutsu et al., 1999) or more elaborate algorithms. The work of Chueh and Lu (2012) is based on p-scores and that of Liang et al. (1998) guides search with Shannon's mutual information. Genetic algorithms are used by Saez-Rodriguez et al. (2009) and Ghaffarizadeh et al. (2017). Linear programming is the basis of Tarissan et al. (2008). The methods of Ostrowski et al. (2016) and Corblin et al. (2012), are based on Answer Set Programming (Brewka et al., 2011). Algebraic methods use reductions (of polynomials over a finite field) modulo an ideal of *vanishing polynomials*.

Approaches based on temporal logic (sometimes augmented with constraints), such as Calzone et al. (2006b) and Mateus et al. (2007), normally employ Model Checking (Clarke et al., 1999). Model Checking, in turn, is often based on symbolic approaches: BDDs and SAT solvers. Biocham (Calzone et al., 2006b) and SMBioNet (Bernot et al., 2004; Khalis et al., 2009; Richard et al., 2012) use a model checker as part of a generate-and-test method.

Having classified various approaches, we now mention a work similar to ours in spirit: Pal et al. (2005). These authors also propagate fixed-point constraints onto the truth tables of the update functions of each variable (molecule species). There is, however, no search technique, other than randomly giving values to the remaining entries of such truth tables. There is a random assignment of values to such entries, and a check for unwanted attractors. Neither is there a formal definition of regulation.

In contrast with the logical approach of our work, we devote our attention now to an *algebraic* approach to the problem of inference (reverse engineering) of Boolean networks. Instead of using a Boolean network to model the dynamics of a gene network, the algebraic approach (Laubenbacher and Stigler, 2004; Jarrah et al., 2007; Veliz-Cuba, 2012) uses a polynomial dynamical system *F*: *S*^*n*^ → *S*^*n*^ where *S* has the structure of a finite field (ℤ/*p* for an appropriate prime number *p*). The first benefit of this algebraic approach is that each component of *F* can be expressed by a polynomial (in *n* variables with coefficients in *S*) such that the degree of each variable is at most equal to the cardinality of the field *S*.

Following a computational algebra approach, in a framework of modeling with polynomial dynamical systems, Laubenbacher and Stigler (2004) propose a reverse-engineering algorithm. This algorithm takes as input a time series $s_{1},\ldots ,s_{m}\in S^{n}$ of network states (where *S* is a finite field), and produces as output a polynomial dynamical system $F=\left(F_{1},\ldots ,F_{n}\right):S^{n}\to S^{n}$ such that ∀*i* ∈ {1, …, *n*}: *F*_*i*_ ∈ *S*[*x*_1_, …, *x*_*n*_] and *F*_*i*_(*s*_*j*_) = *s*_*j*+1,*i*_ for *j* ∈ {1, …, *m*}. An advantage of the algebraic approach of this algorithm is that there exists a well-developed theory of algorithmic polynomial algebra, with a variety of procedures already implemented, supporting the implementation task. The time complexity of this algorithm is *quadratic* in the number of variables (*n*) and *exponential* in the number of time points (*m*).

Comparing *Griffin* with the reverse-engineering algebraic algorithms proposed by Laubenbacher and Stigler (2004) and Veliz-Cuba (2012), we found three basic differences. (1) Algebraic algorithms can handle discrete multi-valued variables, while *Griffin* only handles Boolean (two-valued) variables. Multi-valued variables give more flexibility and detail to the modeling process, but Boolean variables (of Boolean networks) lead to simpler models (see section 1). (2) The input of the algebraic algorithms, typically time series, provides *simple* information coming directly from experimental measurements, while input of *Griffin*, R-graphs and *Griffin* queries, provides *structured* information allowing a more precise specification of the required Boolean network. (3) The algebraic algorithm of Veliz-Cuba (2012) uses a formal definition of regulation, but this definition does not match the definition of regulation used by *Griffin*. While Griffin allows for R-regulations based on Boolean combinations of positive and negative regulations, Veliz-Cuba (2012) uses regulations restricted to *unate* functions *h* such that, for all variable *x*: *h* does not depend on *x*, or *h* depends positively on *x*, or *h* depends negatively on *x*.

Finally, we observe that sometimes results might have been reported overoptimistically. There have been some doubts cast upon the effectiveness of a number of methods of inference of network dynamics (Wimburly et al., 2003), especially those based on more general-purpose learning methods. It is therefore important to establish tests such as the dream challenges (Stolovitzky et al., 2007) emphasizing reproducibility.

### 3.3. Future work

Both *Griffin* and model checkers are based on symbolic search-algorithms, either SAT solvers or BDDs. Hence, a natural possibility to consider is the use of an off-the-shelf model checker as a search mechanism for *Griffin*. Model checkers take as input a temporal-logic formula. We did not find any advantage, however, in using a temporal logic, such as computation-tree logic (CTL) or linear-time logic (LTL), for expressing regulation over ordinary Boolean logic. Once a problem is expressed in Boolean logic, a SAT solver is precisely a method for finding a solution to such a problem. Hence, we disposed of temporal logic and directly expressed the regulation graph in Boolean logic.

Lacking a temporal logic makes the regulation graph the main means of communication of the user with *Griffin*. This suggested enriching the concept of a regulation graph as much as possible, which resulted in our generalization: the R-graph. In addition, the value of a number of parameters can be established with *Griffin*'s query language. Nevertheless, as shown in our case studies, from a Biology point of view, it is easy to fall into situations where *Griffin*'s query language is not expressive enough. In such situations, the user can employ *Griffin*'s API, thus bypassing the query language. Moreover, *Griffin*'s query language (apart from the R-graph, which can be viewed as a shorthand of a Boolean-logic formula) does not have a logical basis. Therefore, compared with a model checker, it would be desirable that *Griffin* have a temporal logic.

Temporal logic has been shown to be effective for the analysis of Boolean networks (Arellano et al., 2011; Carrillo et al., 2012; Klarner and Siebert, 2015). At the same time, our case studies have shown that clause learning could be effective in pruning the search space. This suggests combining Model Checking with clause learning. Much more complex queries than are currently possible could be formulated to *Griffin* in this manner. This is, therefore, an important avenue of research.

Another possibility for future research would explore the combination of *Griffin* with different approaches of Boolean network inference. If the search space is too large for *Griffin* to give useful solutions because of exhausting available resources, *Griffin* could incorporate algorithms based on other approaches, such as genetic algorithms, through its API.

Yet another direction of future work would extend *Griffin* to include either (a) biological information relating species with each other, such as protein complexes, thus reducing the size of the search space, or (b) partially defined Boolean functions for a particular gene.

Finally, we mention an important improvement that would allow the computation and the output of *sets* of Boolean networks instead of a single network at a time. Currently, the SAT solver employed by *Griffin* returns one assignment of *all* the variables appearing in the Boolean formula representing the R-graph together with the constraints (i.e., a “minterm”) at a time. By contrast, it would be more useful to have a SAT solver returning a set of assignments represented by a partial assignment (i.e., a term that is not necessarily a minterm). This would allow *Griffin*, in turn, to output sets of Boolean networks.

## 4. Materials and methods

This section gives definitions and fixes the notation employed in *Griffin*'s algorithms.

### 4.1. General notation

ℕ^+^ is *the set of positive natural numbers*. Unless differently stated, *we assume that n* ∈ ℕ^+^. ${\mathbb{N}}_{n}^{+}$ is an *initial segment* of ℕ^+^, ${\mathbb{N}}_{n}^{+}=\left\{x\in {\mathbb{N}}^{+}|x\leq n\right\}$. 𝔹 = {0, 1} is a set of *Boolean values*. If *b* ∈ 𝔹, then *b*′ is the complement of *b*. If *x* ∈ 𝔹^*n*^, we say that *x* is a *state* and *x*_*i*_ denotes the *i*-th *component* of *x*. We sometimes omit parentheses and commas when writing a state. If *f*:*X*→*X*, and *k* ∈ ℕ^+^, we write *f*^*k*^ to denote the iterated composition of *f* with itself, i.e., *f*^1^ = *f* and *f*^*n*+1^ = *f* ◦ *f*^*n*^. If *v* ∈ 𝔹^*k*^, $p\in {\left({\mathbb{N}}_{n}^{+}\right)}^{k}$, and *p*_*i*_ ≠ *p*_*j*_ for *i* ≠ *j*, then *x*[*v*/*p*] is the state resulting from replacing, for all $i\in {\mathbb{N}}_{k}^{+}$, the *p*_*i*_-th component of *x* by *v*_*i*_. We denote as *x* ~ *i* the vector resulting from replacing the *i*-th *component* of *x* with the complement of *x*_*i*_, that is, $x\sim i=x\left[x_{i}^{\prime }/i\right]$. Given *k* < *n*, *v* ∈ 𝔹^*k*^, and $p\in {\left({\mathbb{N}}_{n}^{+}\right)}^{k}$, 𝔹^*n*^[*v*/*p*] is a *subspace* of 𝔹^*n*^ defined by 𝔹^*n*^[*v*/*p*] = {*x*[*v*/*p*]∣*x* ∈ 𝔹^*n*^}. Alternatively, we describe subspaces by means of strings of zeros, ones, and asterisks used as wildcards indicating the free (unknown value) components. For example, 𝔹^6^[(0, 1, 0)/(1, 4, 6)] can be written simply as 0^**^1^*^0.

### 4.2. Synchronous boolean networks, regulation graphs, and state graphs

*Definition 1*. We define a *synchronous Boolean network with n components* as a function *f*:𝔹^*n*^→𝔹^*n*^. The *i-th component of f* is a function $f_{i}:{𝔹}^{n}\to 𝔹$ such that *f*_*i*_(*x*) = *f*(*x*)_*i*_. We use *BN* to denote the class of Boolean networks with *n* components.

To relate a synchronous Boolean network with a molecular network dynamics, we interpret each component of a state *x* as representing the activation state of a particular variable denoting a molecule included in the network (which can be a gene, a protein, a hormone, for example). Given a set of variables *V*, we use a bijective function $v:{\mathbb{N}}_{n}^{+}\to V$ to relate the components of a state *x* with their respective variables. The molecule represented by the variable *v*(*i*), also denoted *v*_*i*_, is said to be *active* if *x*_*i*_ = 1 and *inactive* otherwise.

*Definition 2*. If $p\in {\left({\mathbb{N}}_{n}^{+}\right)}^{k}$, and *v* ∈ 𝔹^*k*^ with $k\in {\mathbb{N}}_{n}^{+}$, *the mutation of f* by *p* = *v* is defined as *f*^*p* = *v*^: 𝔹^*n*^ → 𝔹^*n*^, where, for all *x* ∈ 𝔹^*n*^ and $j\in {\mathbb{N}}_{k}^{+}$, $f_{p_{j}}^{p=v}\left(x\right)=v_{j}$, and $f_{i}^{p=v}\left(x\right)=f_{i}\left(x\right)$ if $i\in {\mathbb{N}}_{n}^{+}-\left\{p_{1},\ldots ,p_{k}\right\}$. We say that *f*^*p* = *v*^ is a *single-point mutation* of *f* if *k* = 1, and a *multi-point mutation* if *k* > 1.

*Definition 3*. (Naldi et al., 2007; Richard et al., 2012; Mori and Mochizuki, 2017). A *positive regulation* between variables *v*_*i*_ and *v*_*j*_ is a function *R*^+^: *V* × *BN* × *V* → 𝔹 such that

$$R^{+}(v_{i},f,v_{j})=\{\begin{array}{c} 1\text{ if }\exists x\in {𝔹}^{n}:f_{j}(x)\neq f_{j}(x\sim i)\text{ and }x_{i}=f_{j}(x)\\ 0\text{ otherwise} \end{array}$$

Similarly, a *negative regulation* between *v*_*i*_ and *v*_*j*_ is a function *R*^−^: *V* × *BN* × *V* → 𝔹 such that

$$R^{-}(v_{i},f,v_{j})=\{\begin{array}{c} 1\text{ if }\exists x\in {𝔹}^{n}:f_{j}(x)\neq f_{j}(x\sim i)\text{ and }x_{i}\neq f_{j}(x)\\ 0\text{ otherwise} \end{array}$$

*Definition 4*. We say that *G* is a *regulation graph* if *G* = (*V, I*^+^, *I*^−^), where: *V* is the set of vertices, *I*^+^ ⊆ *V* × *V* is the set of *positive regulations*, and *I*^−^ ⊆ *V* × *V* is a set of *negative regulations*. If *i* ∈ *I*^+^ ∩ *I*^−^, we say that *i* is an *ambiguous regulation*.

*Definition 5*. The regulation graph of *f* is $G_{f}=\left(V,I^{+},I^{-}\right)$ where:

- $\left(v_{i},v_{j}\right)\in I^{+}$ iff $R^{+}\left(v_{i},f,v_{j}\right)=1$.
- $\left(v_{i},v_{j}\right)\in I^{-}$ iff $R^{-}\left(v_{i},f,v_{j}\right)=1$.

Intuitively, the regulation graph of *f*, *G*_*f*_, describes the structure of *f* and its interpretation as a molecular network where edges represent molecular regulations. Note that *G*_*f*_ may have both a positive and a negative regulation from *j* to *i*. Observe that whereas a Boolean network *f* has a unique regulation graph, a regulation graph may have more than one Boolean network.

*Definition 6*. Given a regulation graph *G*, we say that *f* is a *satisfying Boolean network* of *G*, denoted by *f* ⊨ *G*, if *G* = *G*_*f*_. The set of all satisfying Boolean networks of *G* is denoted by *F*_*G*_ = {*f*∣*f* ⊨ *G*}.

*Definition 7*. The *state graph* of a Boolean network *f* is the graph ${Ĝ}_{f}=\left({𝔹}^{n},\left\{\left(x,f\left(x\right)\right)|x\in {𝔹}^{n}\right\}\right)$.

*Definition 8*. We say that ω ⊆ 𝔹^*n*^ is an *attractor* of *f* if ω is a terminal strongly connected component of Ĝ_*f*_ (Ruet, 2017). That is, ω is an attractor if ω is strongly connected (each state in ω is reachable from any other state in ω) and no state or edge of Ĝ_*f*_ can be added to ω without causing ω to be no longer strongly connected. In particular, ω is a *fixed point* (or *stationary state*) of *f* if |ω| = 1, it is a *cyclic attractor* otherwise. The *size k* of an attractor is defined as its cardinality. The *set of attractors* of *f* is denoted as A(*f*). The *set of fixed points* of *f* is denoted as FP(*f*). The *set of cyclic attractors* of *f* is denoted as CA(*f*). If ω ∈ A(*f*), the *basin of attraction* of ω is $\mathrm{BA}\left(\omega \right)=\left\{x|x\in {𝔹}^{n}\text{and}\exists k\in {\mathbb{N}}_{2^{n}-1}^{+}:f^{k}\left(x\right)\in \omega \right\}$.

### 4.3. Boolean-network inference

#### 4.3.1. Constrained-regulation graphs and R-graphs

Before formally introducing a Boolean network query, we provide additional definitions that will allow us to express partial knowledge of regulation graphs.

*Definition 9*. A *constrained-regulation graph G* = (*V, I*) is a labeled graph over the set of variables *V*, where *I* is a set of *constrained regulations I* ⊆ *V* × *L* × *V*. Labels *L* = {*g*∣ *g*: *V* × *BN* × *V* → 𝔹} are *regulation constraints* limiting the acceptable Boolean networks.

*Definition 10*. We say that *f satisfies* a constrained-regulation graph *G* = (*V, I*), *f* ⊨ *G*, if ∀*u, v*: (*u, g, v*) ∈ *I* implies *g*(*u, f, v*) = 1. The set of all satisfying Boolean networks of *G* is denoted by *F*_*G*_ = {*f* ∣ *f* ⊨ *G*}.

It can be proved that given a regulation graph *G* = (*V, I*^+^, *I*^−^), if *G*′ = (*V, I*) is defined in such a way that (*u, g, v*) ∈ *I* iff

- (*u, v*) ∈ *I*^+^ implies *g*(*u, f, v*) = *R*^+^(*u, f, v*) and
- (*u, v*) ∈ *I*^−^ implies *g*(*u, f, v*) = *R*^−^(*u, f, v*),

then ∀*f* ∈ *BN* : *f* ⊨ *G* iff *f* ⊨ *G*′.

*Definition 11*. An *R-graph* is a *constrained-regulation graph G* = (*V, I*) where the constraints are Boolean combinations of positive and negative regulations, that is, ∀(*u, g, v*) ∈ *I*: ∃*h*: 𝔹^2^ → 𝔹 such that *g*(*u, f, v*) = *h*(*R*^+^(*u, f, v*), *R*^−^(*u, f, v*)) with *f* ∈ *BN*. Edges of an R-graph are called *R-regulations*.

Table 1 shows the list of possible R-regulations for an R-graph. An example of an R-graph is shown in Figure 1. R-graphs are of special interest because they can be viewed as generalizations of ordinary regulation graphs, that are able to express hypotheses and incomplete knowledge about the dependencies between network variables.

*Definition 12*. Given a regulation graph $G_{1}=\left(V,I^{+},I^{-}\right)$ and a constrained-regulation graph *G*_2_ = (*V, I*), we say that *G*_1_ is an *instantiation* of *G*_2_ if there exists *f* ∈ *BN* such that *f* ⊨ *G*_1_ and *f* ⊨ *G*_2_. The set of all instantiations of *G* is denoted as *G*_*G*_.

Instantiations of constrained-regulation graphs are regulations graphs that share the same satisfying networks, Figure 1 shows an example of the set of all instantiations of an R-graph.

#### 4.3.2. Network constraint problems and queries

*Definition 13*. A *network constraint c* is a Boolean function on the class of Boolean networks, *c*:*BN* → 𝔹.

The previous definition is a particular case of a Boolean constraint of arity *k* as defined in Creignou et al. (2001) and Dantsin and Hirsch (2009). In our case *k* = *n*2^*n*^, given that we have 2^*n*^ states in 𝔹^*n*^ and for each state *x* there are *n* components specifying the next state *F*(*x*).

Examples of meaningful network constraints include, but are not limited to:

1. Network equality: *c*(*f*) = 1 iff *f* = *f*_0_ for *f*_0_ ∈ *BN*.
2. Network membership: *c*(*f*) = 1 iff *f* ∈ *F*_0_ for *F*_0_ ⊂ *BN*.
3. Attractor equality: *c*(*f*) = 1 iff *A*(*f*) = *S*_0_ for $S_{0}\subset P\left(𝔹\right)$.
4. Steady-state inclusion: *c*(*f*) = 1 iff FP(*f*) ⊇ 𝔹_0_ for $B_{0}\subset {𝔹}^{n}$.
5. Steady-state prohibitions: *c*(*f*) = 1 iff *x*_0_ ∉ FP(*f*) for $x_{0}\in {𝔹}^{n}$.
6. Fixed-point equality after mutation: *c*(*f*) = 1 iff $\mathrm{FP}\left(f^{p=v}\right)=B_{0}$ for $p\in {\left({\mathbb{N}}_{n}^{+}\right)}^{k}$, *v* ∈ 𝔹^*k*^, $k\in {\mathbb{N}}_{n}^{+}$ and $B_{0}\subset {𝔹}^{n}$.
7. Fixed-point inclusion after mutation:*c*(*f*) = 1 iff $\mathrm{FP}\left(f^{p=v}\right)\subseteq B_{0}$ for $p\in {\left({\mathbb{N}}_{n}^{+}\right)}^{k}$, *v* ∈ 𝔹^*k*^, $k\in {\mathbb{N}}_{n}^{+}$ and $B_{0}\subset {𝔹}^{n}$.
8. State transition presence:
  *c*(*f*) = 1 iff *f*(*x*_0_) = *y*_0_ for $x_{0},y_{0}\in {𝔹}^{n}$.

Note that *network constraints* are different from the *constraint regulations* of definition 9. As can be seen in the previous listing, the former refer to general properties of *f*, the Boolean network to be inferred, whereas the latter refer to specific properties of the regulations between pairs of variables, for example restricting a regulation to be a positive regulation.

*Definition 14*. Given a constrained-regulation graph *G* = (*V, I*) and a set of network constraints *C*, we say that *P* is a *network constraint problem (NCP) P* = (*G, C*), where *C* = {*c*_1_, …, *c*_*m*_} is a set of network constraints. We say that *f* ∈ *BN* is a *solution* of *P*, denoted as *f* ⊨ *P*, if *f* ⊨ *G* and for all *c* ∈ *C*, *c*(*f*) = 1. The set of all solutions of *P* is *F*_*P*_.

*Definition 15*. Given a network-constraint problem *P* = (*G, C*), we consider two possible queries over the set of solutions of *P*:

- k-search queries, denoted search_*k*_(*P*): find any *F*_0_ ⊆ *BN* such that ∀*f* ∈ *F*_0_:*f* ⊨ *P* and |*F*| = min(*k*, |*F*_*P*_|),
- All-search queries, denoted search(*P*): find all *f* such that *f* ⊨ *P*.

*A query Q over a set of solutions of P is denoted as Q(P). The class of queries is symbolized by Q. The set of solutions satisfying a query Q is denoted as F_Q_. We call Griffin queries the queries implemented in Griffin, a subset of the class Q (see http://turing.iimas.unam.mx/griffin/guide.html#examplesfile)*.

*Definition 16*. Given a query *Q*(*P*) with *P* = (*G, C*), a *query splitting* is a function s:Q→P(Q) such that

- ∀*q*(*g, C*) ∈ *s*(*Q*):*f* ⊨ *g* → *f* ⊨ *G* and
- *f* ⊨ *G* → ∃*q*(*g, C*) ∈ *s*(*Q*): *f* ⊨ *g*.

## Author contributions

DR conceived the project. DR, EA, and SM designed the research. SM developed *Griffin* and performed the research. EA designed and directed the case studies. MC, DR, and SM developed the formal definitions and theoretical results. SM and MC prepared tables and figures. All authors wrote the manuscript.

### Conflict of interest statement

The authors declare that the research was conducted in the absence of any commercial or financial relationships that could be construed as a potential conflict of interest.

## References

[B1] Akutsu T. Miyano S. Kuhara S. (1999). Identification of genetic networks from a small number of gene expression patterns under the Boolean network model, in Pacific Symposium on Biocomputing, Vol. 4 (Hawaii), 17–28. 10.1142/9789814447300_0003 10380182

[B2] Albert R. Othmer H. G. (2003). The topology of the regulatory interactions predicts the expression pattern of the segment polarity genes in *Drosophila melanogaster*. J. Theor. Biol. 223, 1–18. 10.1016/S0022-5193(03)00035-3 12782112 PMC6388622

[B3] Alvarez-Buylla E. R. Benítez M. Corvera-Poiré A. Chaos Cador Á. de Folter S. Gamboa de Buen A.. (2010). Flower development. Arabidopsis Book 8:e0127. 10.1199/tab.0127 22303253 PMC3244948

[B4] Arellano G. Argil J. Azpeitia E. Benítez M. Carrillo M. Góngora P.. (2011). “Antelope”: a hybrid-logic model checker for branching-time Boolean GRN analysis. BMC Bioinformatics 12:490. 10.1186/1471-2105-12-490 22192526 PMC3316443

[B5] Azpeitia E. Benítez M. Vega I. Villarreal C. Alvarez-Buylla E. R. (2010). Single-cell and coupled GRN models of cell patterning in the *Arabidopsis thaliana* root stem cell niche. BMC Syst. Biol. 4:1. 10.1186/1752-0509-4-134 20920363 PMC2972269

[B6] Azpeitia E. Davila-Velderrain J. Villarreal C. Alvarez-Buylla E. R. (2014). Chapter 26: Gene regulatory network models for floral organ determination, in Flower Development: Methods and Protocols, Methods in Molecular Biology, Vol. 1110, eds Riechmann J. L. Wellmer F. (New York, NY: Springer), 441–469. 10.1007/978-1-4614-9408-9_26 24395275

[B7] Azpeitia E. Muñoz S. González-Tokman D. Martínez-Sánchez M. E. Weinstein N. Naldi A. (2017). The combination of the functionalities of feedback circuits is determinant for the attractors' number and size in pathway-like Boolean networks. Sci. Rep. 7:42023 10.1038/srep42023 28186191 PMC5301197

[B8] Azpeitia E. Weinstein N. Benítez M. Mendoza L. Alvarez-Buylla E. R. (2013). Finding missing interactions of the *Arabidopsis thaliana* root stem cell niche gene regulatory network. Front. Plant Sci. 4:110. 10.3389/fpls.2013.00110 23658556 PMC3639504

[B9] Barman S. Kwon Y.-K. (2017). A novel mutual information-based Boolean network inference method from time-series gene expression data. PLoS ONE 12:e0171097. 10.1371/journal.pone.0171097 28178334 PMC5298315

[B10] Berestovsky N. Nakhleh L. (2013). An evaluation of methods for inferring Boolean networks from time-series data. PLoS ONE 8:e66031. 10.1371/journal.pone.0066031 23805196 PMC3689729

[B11] Bernot G. Comet J.-P. Richard A. Guespin J. (2004). Application of formal methods to biological regulatory networks: extending Thomas' asynchronous logical approach with temporal logic. J. Theor. Biol. 229, 339–347. 10.1016/j.jtbi.2004.04.003 15234201

[B12] Bornholdt S. (2001). Modeling genetic networks and their evolution: a complex dynamical systems perspective. Biol. Chem. 382, 1289–1299. 10.1515/BC.2001.161 11688712

[B13] Bornholdt S. (2005). Systems biology: less is more in modeling large genetic networks. Science 310, 449–451. 10.1126/science.1119959 16239464

[B14] Bornholdt S. (2008). Boolean network models of cellular regulation: prospects and limitations. J. R. Soc. Interface 5, S85–S94. 10.1098/rsif.2008.0132.focus 18508746 PMC2386560

[B15] Brewka G. Eiter T. Truszczyński M. (2011). Answer Set Programming at a glance. Commun. ACM 54, 92–103. 10.1145/2043174.2043195

[B16] Burch J. Clarke E. McMillan K. Dill D. Hwang L. (1992). Symbolic model checking: 10^20^ states and beyond. Inform. Comput. 98, 142–170. 10.1016/0890-5401(92)90017-A

[B17] Calzone L. Chabrier-Rivier N. Fages F. Soliman S. (2006a). Machine learning biochemical networks from temporal logic properties, in Transactions on Computational Systems Biology VI, Lecture Notes in Bioinformatics No. 4220, ed Plotkin G. (Berlin; Heidelberg: Springer), 68–94.

[B18] Calzone L. Fages F. Soliman S. (2006b). BIOCHAM: an environment for modeling biological systems and formalizing experimental knowledge. Bioinformatics 22, 1805–1807. 10.1093/bioinformatics/btl172 16672256

[B19] Carrillo M. Góngora P. A. Rosenblueth D. A. (2012). An overview of existing modeling tools making use of model checking in the analysis of biochemical networks. Front. Plant Sci. 3:155. 10.3389/fpls.2012.00155 22833747 PMC3400939

[B20] Chabrier-Rivier N. Chiaverini M. Danos V. Fages F. Schächter V. (2004). Modeling and querying biomolecular interaction networks. Theor. Comput. Sci. 325, 25–44. 10.1016/j.tcs.2004.03.063

[B21] Chueh T.-H. Lu H. H.-S. (2012). Inference of biological pathway from gene expression profiles by time delay Boolean networks. PLoS ONE 7:e42095. 10.1371/journal.pone.0042095 22952589 PMC3432056

[B22] Clarke E. M. Grumberg O. Peled D. A. (1999). Model Checking. Cambridge, MA; London: MIT Press.

[B23] Corblin F. Fanchon E. Trilling L. Chaouiya C. Thieffry D. (2012). Automatic inference of regulatory and dynamical properties from incomplete gene interaction and expression data, in Information Processing in Cells and Tissues (Berlin; Heidelberg), 25–30. 10.1007/978-3-642-28792-3_4

[B24] Creignou N. Khanna S. Sudan M. (2001). Complexity Classifications of Boolean Constraint Satisfaction Problems. Philadelphia, PA: SIAM 10.1137/1.9780898718546

[B25] Dantsin E. Hirsch E. A. (2009). Worst-case upper bounds, in Handbook of Satisfiability, Vol. 185, eds Biere A. Heule M. van Maaren H. Walsh T. (IOS Press), 403–424. 10.3233/978-1-58603-929-5-403

[B26] Davidich M. I. Bornholdt S. (2008). Boolean network model predicts cell cycle sequence of fission yeast. PLoS ONE 3:e1672. 10.1371/journal.pone.0001672 18301750 PMC2243020

[B27] D'haeseleer P. Liang S. Somogyi R. (2000). Genetic network inference: from co-expression clustering to reverse engineering. Bioinformatics 16, 707–726. 10.1093/bioinformatics/16.8.707 11099257

[B28] Drinnan A. N. Crane P. R. Hoot S. B. (1994). Patterns of floral evolution in the early diversification of non-magnoliid dicotyledons (eudicots), in Early Evolution of Flowers. Plant Systematics and Evolution Supplement 8, Vol. 8, eds Endress P. K. Friis E. M. (Vienna: Springer), 93–122.

[B29] Dubrova E. Teslenko M. (2011). A SAT-based algorithm for finding attractors in synchronous Boolean networks. IEEE/ACM Trans. Comput. Biol. Bioinformatics (TCBB) 8, 1393–1399. 10.1109/TCBB.2010.20 21778527

[B30] Espinosa-Soto C. Padilla-Longoria P. Alvarez-Buylla E. (2004). A gene regulatory network model for cell-fate determination during *Arabidopsis thaliana* flower development that is robust and recovers experimental gene expression profiles. Plant Cell 16, 2923–2939. 10.1105/tpc.104.021725 15486106 PMC527189

[B31] Fages F. Soliman S. (2008). Abstract interpretation and types for systems biology. Theor. Comput. Sci. 403, 52–70. 10.1016/j.tcs.2008.04.024

[B32] Fages F. Soliman S. Chabrier-Rivier N. (2004). Modelling and querying interaction networks in the biochemical abstract machine BIOCHAM. J. Biol. Phys. Chem. 4, 64–73. 10.4024/2040402.jbpc.04.02

[B33] Fisher J. Henzinger T. A. (2007). Executable cell biology. Nat. Biotechnol. 25, 1239–1249. 10.1038/nbt1356 17989686

[B34] Franco J. Martin J. (2009). A history of satisfiability, in Handbook of Satisfiability, vol. 185, eds Biere A. Heule M. van Maaren H. Walsh T. (Amsterdam; Berlin; Oxford; Tokyo; Washington, DC: IOS Press), 3–74.

[B35] García-Gómez M. L. Azpeitia E. Álvarez Buylla E. R. (2017). A dynamic genetic-hormonal regulatory network model explains multiple cellular behaviors of the root apical meristem of *Arabidopsis thaliana*. PLoS Comput. Biol. 13:e1005488. 10.1371/journal.pcbi.1005488 28426669 PMC5417714

[B36] Garg A. Di Cara A. Xenarios I. Mendoza L. De Micheli G. (2008). Synchronous versus asynchronous modeling of gene regulatory networks. Bioinformatics 24, 1917–1925. 10.1093/bioinformatics/btn336 18614585 PMC2519162

[B37] Gershenson C. (2002). Classification of random boolean networks in Standish, in Artificial Life VIII: Proceedings of the Eighth International Conference on Artificial Life, eds Standish R. K. Bedau M. A. Abbass H. A. (Sydney, NSW: MIT Press), 1–8.

[B38] Ghaffarizadeh A. Podgorski G. J. Flann N. S. (2017). Applying attractor dynamics to infer gene regulatory interactions involved in cellular differentiation. Biosystems 155, 29–41. 10.1016/j.biosystems.2016.12.004 28254369

[B39] Haider S. Pal R. (2012). Boolean network inference from time series data incorporating prior biological knowledge. BMC Genomics 13:S9. 10.1186/1471-2164-13-S6-S9 23134816 PMC3481452

[B40] Han S. Wong R. K. Lee T. C. Shen L. Li S.-Y. R. Fan X. (2014). A full bayesian approach for boolean genetic network inference. PloS ONE 9:e115806. 10.1371/journal.pone.0115806 25551820 PMC4281059

[B41] Hecker M. Lambeck S. Toepfer S. van Someeren E. Guthke R. (2009). Gene regulatory network inference: data integration in dynamic models—a review. BioSystems 96, 86–103. 10.1016/j.biosystems.2008.12.004 19150482

[B42] Hickman G. J. Hodgman T. C. (2009). Inference of gene regulatory networks using Boolean-network inference methods. J. Bioinform. Comput. Biol. 7, 1013–1029. 10.1142/S0219720009004448 20014476

[B43] Huang S. (1999). Gene expression profiling, genetic networks, and cellular states: an integrating concept for tumorigenesis and drug discovery. J. Mol. Med. 77, 469–480. 10.1007/s001099900023 10475062

[B44] Jarrah A. S. Laubenbacher R. Stigler B. Stillman M. (2007). Reverse-engineering of polynomial dynamical systems. Adv. Appl. Math. 39, 477–489. 10.1016/j.aam.2006.08.004

[B45] Karlebach G. Shamir R. (2008). Modelling and analysis of gene regulatory networks. Nat. Rev. Mol. Cell Biol. 9, 770–780. 10.1038/nrm2503 18797474

[B46] Kauffman S. (1969). Homeostasis and differentiation in random genetic control networks. Nature 224, 177–178. 10.1038/224177a0 5343519

[B47] Khalis Z. Comet J.-P. Richard A. Bernot G. (2009). The SMBioNet method for discovering models of gene regulatory networks, in Focus on Bioinformatics. Genes, Genomes and Genomics Vol. 3 (Special Issue 1). Global Science Books 2009, ed Mansour A. 15–22. Available online at: http://www.globalsciencebooks.info/Online/GSBOnline/OnlineGGG_3_SI1.html

[B48] Klarner H. Bockmayr A. Siebert H. (2014). Computing symbolic steady states of boolean networks, in Cellular Automata. ACRI 2014. Lecture Notes in Computer Science, Vol. 8751, eds Wąs J. Sirakoulis G. C. Bandini S. (Cham: Springer), 561–570.

[B49] Klarner H. Siebert H. (2015). Approximating attractors of boolean networks by iterative ctl model checking. Front. Bioeng. Biotechnol. 3:130. 10.3389/fbioe.2015.00130 26442247 PMC4562258

[B50] La Rota C. Chopard J. Das P. Paindavoine S. Rozier F. Farcot E.. (2011). A data-driven integrative model of sepal primordium polarity in *Arabidopsis*. Plant Cell 23, 4318–4333. 10.1105/tpc.111.092619 22198150 PMC3269868

[B51] Lähdesmäki H. Shmulevich I. Yli-Harja O. (2003). On learning gene regulatory networks under the Boolean network model. Mach. Learn. 52, 147–167. 10.1023/A:1023905711304

[B52] Laubenbacher R. Stigler B. (2004). A computational algebra approach to the reverse engineering of gene regulatory networks. J. Theor. Biol. 229, 523–537. 10.1016/j.jtbi.2004.04.037 15246788

[B53] Layek R. K. Datta A. Dougherty E. R. (2011). From biological pathways to regulatory networks. Mol. Biosyst. 7, 843–851. 10.1039/C0MB00263A 21161088

[B54] Li F. Long T. Lu Y. Ouyang Q. Tang C. (2004). The yeast cell-cycle network is robustly designed. Proc. Natl. Acad. Sci. U.S.A. 101, 4781–4786. 10.1073/pnas.0305937101 15037758 PMC387325

[B55] Liang S. Fuhrman S. Somogyi R. (1998). REVEAL, a general reverse engineering algorithm for inference of genetic network architectures. Pac. Symp. Biocomput. 3, 18–29. 9697168

[B56] Markowetz F. Spang R. (2007). Inferring cellular networks–a review. BMC Bioinformatics 8:S5. 10.1186/1471-2105-8-S6-S5 17903286 PMC1995541

[B57] Mateus D. Gallois J.-P. Comet J.-P. Gall P. L. (2007). Symbolic modeling of genetic regulatory networks. J. Bioinform. Comput. Biol. 5, 627–640. 10.1142/S0219720007002850 17636866

[B58] Mendoza L. (2006). A network model for the control of the differentiation process in Th cells. Biosystems 84, 101–114. 10.1016/j.biosystems.2005.10.004 16386358

[B59] Mendoza L. Alvarez-Buylla E. R. (1998). Dynamics of the genetic regulatory network for *Arabidopsis thaliana* flower morphogenesis. J. Theor. Biol. 193, 307–319. 10.1006/jtbi.1998.0701 9714934

[B60] Mendoza L. Thieffry D. Alvarez-Buylla E. R. (1999). Genetic control of flower morphogenesis in *Arabidopsis thaliana*: a logical analysis. Bioinformatics 15, 593–606. 10.1093/bioinformatics/15.7.593 10487867

[B61] Mori F. Mochizuki A. (2017). Expected number of fixed points in Boolean networks with arbitrary topology. Phys. Rev. Lett. 119:028301. 10.1103/PhysRevLett.119.028301 28753377

[B62] Naldi A. Thieffry D. Chaouiya C. (2007). Decision diagrams for the representation and analysis of logical models of genetic networks, in Computational Methods in Systems Biology. CMSB 2007. Lecture Notes in Computer Science, Vol. 4695, eds Calder M. Gilmore S. (Berlin; Heidelberg: Springer), 15–31.

[B63] Ostrowski M. Paulevé L. Schaub T. Siegel A. Guziolowski C. (2016). Boolean network identification from perturbation time series data combining dynamics abstraction and logic programming. Biosystems 149, 139–153. 10.1016/j.biosystems.2016.07.009 27484338

[B64] Pal R. Ivanov I. Datta A. Bittner M. L. Dougherty E. R. (2005). Generating Boolean networks with a prescribed attractor structure. Bioinformatics 21, 4021–4025. 10.1093/bioinformatics/bti664 16150807

[B65] Richard A. Rossignol G. Comet J.-P. Bernot G. Guespin-Michel J. Merieau A. (2012). Boolean models of biosurfactants production in *Pseudomonas fluorescens*. PLoS ONE 7:e24651. 10.1371/journal.pone.0024651 22303435 PMC3269426

[B66] Rosenblueth D. A. Muñoz S. Carrillo M. Azpeitia E. (2014). Inference of boolean networks from gene interaction graphs using a SAT solver, in Algorithms for Computational Biology. AlCoB 2014. Lecture Notes in Computer Science, Vol. 8542, eds Dediu A. H. Martín-Vide C. Truthe B. (Cham: Springer), 235–246. 10.1007/978-3-319-07953-0_19

[B67] Ruet P. (2017). Negative local feedbacks in Boolean networks. Discrete Appl. Math. 221, 1–17. 10.1016/j.dam.2017.01.001

[B68] Saadatpour A. Albert I. Albert R. (2010). Attractor analysis of asynchronous Boolean models of signal transduction networks. J. Theor. Biol. 266, 641–656. 10.1016/j.jtbi.2010.07.022 20659480

[B69] Saez-Rodriguez J. Alexopoulos L. G. Epperlein J. Samaga R. Lauffenburger D. A. Klamt S.. (2009). Discrete logic modelling as a means to link protein signalling networks with functional analysis of mammalian signal transduction. Mol. Syst. Biol. 5:331. 10.1038/msb.2009.87 19953085 PMC2824489

[B70] Shanahan M. (1997). Solving the Frame Problem. A mathematical investigation of the Common Sense Law of Inertia. Cambridge, MA; London: MIT Press.

[B71] Shmulevich I. Saarinen A. Yli-Harja O. Astola J. (2003). Chapter 11: Inference of genetic regulatory networks via best-fit extensions, in Computational and Statistical Approaches to Genomics (New York, NY; Boston; Dordrecht; London; Moscow: Springer), 197–210.

[B72] Stolovitzky G. Monroe D. Califano A. (2007). Dialogue on reverse-engineering assessment and methods. The DREAM of high-throughput pathway inference. Ann. N.Y. Acad. Sci. 1115, 1–22. 10.1196/annals.1407.021 17925349

[B73] Streck A. Siebert H. (2015). Extensions for LTL model checking of Thomas networks, in Proceedings of the Strasbourg Spring School on Advances in Systems and Synthetic Biology, eds Amar P. Képès F. Norris V. 101–114. Available online at: https://www.researchgate.net/publication/278804124_avances_in_Systems_and_Synthetic_Biology

[B74] Tarissan F. Liberti L. La Rota C. (2008). Network reconstruction: a mathematical programming approach, in European Conference on Complex Systems (ECCS'08) (Jerusalem). Available online at: https://hal.archives-ouvertes.fr/hal-01217842

[B75] Tseitin G. (1968). On the complexity of derivation in propositional calculus, in Studies in Constructive Mathematics and Mathematical Logic, Part 2, ed Slisenko A. (New York, NY; London: Consultants Bureau), 115–125.

[B76] Veliz-Cuba A. (2012). An algebraic approach to reverse engineering finite dynamical systems arising from biology. SIAM J. Appl. Dyn. Syst. 11, 31–48. 10.1137/110828794

[B77] Weinstein N. Ortiz-Gutiérrez E. Muñoz S. Rosenblueth D. A. Álvarez-Buylla E. R. Mendoza L. (2015). A model of the regulatory network involved in the control of the cell cycle and cell differentiation in the *Caenorhabditis elegans* vulva. BMC Bioinformatics 16:1. 10.1186/s12859-015-0498-z 25884811 PMC4367908

[B78] Wimburly F. C. Heiman T. Ramsey J. Glymour C. (2003). Experiments on the accuracy of algorithms for inferring the structure of genetic regulatory networks from microarray expression levels, in International Joint Conference on Artificial Intelligence Workshop (San Francisco, CA).

