# Supplementary material for: Griffin: A Tool for Symbolic Inference of Synchronous Boolean Molecular Networks
Source: Front Genet. 2018 Mar 6;9:39. doi: 10.3389/fgene.2018.00039 (PMC5845696; doi:10.3389/fgene.2018.00039)
Supplement: Supplementary file 1 [file Presentation1.pdf]

Supplementary Material: Griffin: a tool for symbolic inference of synchronous Boolean molecular networks

Stalin Muñoz , Miguel Carrillo , Eugenio Azpeitia , and David A. Rosenblueth

1 QUERY FILES

Query files included in this section were used in the case studies documented in the main article.

```
1 #Name and order of nodes
2 genes={FUL,FT,AP1,EMF1,LFY,AP2,WUS,AG,TFL1,PI,SEP,AP3,UFO}
3 #Regulations,
4 #see figure 20 of Alvarez-Buylla, et al. (2010).
5 #Flower development. The Arabidopsis book/American Society of Plant Biologists, 8.
6 known = {AG->AG, AG-|AP1, AG->AP3, AG->PI, AG-|WUS, AP1-|AG, AP1->AP3, AP1-|FUL, AP1->PI, AP1-|TFL1, AP2-|AG, AP3-|AP1, AP3->AP3, AP3->PI, EMF1-|AG, EMF1-|FT, EMF1-|LFY, EMF1->TFL1, FT->AP1, LFY->AG, LFY->AP1, LFY->AP3, LFY-|EMF1, LFY->PI, LFY->SEP, LFY-|TFL1, PI-|AP1, PI->AP3, PI->PI, SEP->AG, SEP->AP3, SEP->PI, SEP-|WUS, TFL1-|AG, TFL1-|AP1, TFL1-|AP2, TFL1-|FUL, TFL1-|LFY, UFO->AP3, UFO->UFO, WUS->AG, WUS->WUS}
7 #options
8 allow.ambiguity = false
9 allow.additional.states = true
10 allow.additional.cycles = false
11 allow.hypotheses = false
12 block.steady.a.posteriori = false
13 divide.query.by.topology = false
```

Listing 1: *Griffin* query file representing the constraint of known regulatory interactions of Alvarez-Buylla et al. (2010) model depicted in Fig. 4a in the main text. The query first specifies the list and order of genes in line 2 (the order of the genes is important for defining constraints on network states such as fixed point attractors). Line 6 specifies the list of known interactions. The sign of an interaction is declared using infix notation, −> for positive interactions and −| for negative interactions. At the end of the query there is a list of options, the query asks for the following: a) no ambiguous regulations (line 8), b) any combination of fixed point attractors (line 9), and c) no cyclic attractors (line 10).

```
1  #Name and order of nodes
2  genes={FUL,FT,AP1,EMF1,LFY,AP2,WUS,AG,TFL1,PI,SEP,AP3,UFO}
3  #Regulations,
4  #see figure 20 of Alvarez-Buylla, et al. (2010).
5  #Flower development. The Arabidopsis book/American Society of Plant Biologists, 8.
6  known = {AG->AG, AG-|AP1, AG->AP3, AG->PI, AG-|WUS, AP1-|AG, AP1->AP3, AP1-|FUL, AP1->PI, AP1-|TFL1, AP2-|AG, AP3-|AP1, AP3->AP3, AP3->PI, EMF1-|AG, EMF1-|FT, EMF1-|LFY, EMF1->TFL1, FT->AP1, LFY->AG, LFY->AP1, LFY->AP3, LFY-|EMF1, LFY->PI, LFY->SEP, LFY-|TFL1, PI-|AP1, PI->AP3, PI->PI, SEP->AG, SEP->AP3, SEP->PI, SEP-|WUS, TFL1-|AG, TFL1-|AP1, TFL1-|AP2, TFL1-|FUL, TFL1-|LFY, UFO->AP3, UFO->UFO, WUS->AG, WUS->WUS,FUL->LFY,AP1->LFY, AP2-|TFL1}
7  #steady states
8  fixed-points()={0001000010000,0001000010001,0001001010000,0001001010001, 0110110000100,0110110001110,0110110001111,1100110101100,1100110101110, 1100110101111}
9  #options
10 allow.ambiguity = false
11 allow.additional.states = false
12 allow.additional.cycles = false
13 allow.hypotheses = false
14 block.steady.a.posteriori = true
15 divide.query.by.topology = false
```

Listing 2: *Griffin* query file representing the constraint of known regulatory interactions and known set of ten fixed-point attractors. The set of known fixed-point attractors for the model defined in Fig. 4a in the main text is specified in line 8. The expression profiles are represented as a sequence of ones and zeros following the ordering of the genes specified in line 2. Any additional attractor not in the specified set is to be blocked (line 11) by *Griffin* using *a posteriori* refinement (line 14).

```
1  #Name and order of nodes
2  genes={AGO1, AGO10, AGO7, ANT, ARF4, AS1, AS2, ETT, FIL, KAN1, miR165, miR390, REV, TAS3siRNA, AGO1_miR165, AGO7_miR390, AS1_AS2, AUXINh, CKh, GTE6, IPT5}
3  #All known regulations
4  known={AGO1->AGO1_miR165,AGO7->AGO7_miR390,AS1->AS1_AS2,AS2->AS1_AS2,KAN1-|AS2,miR165->AGO1_miR165,miR390->AGO7_miR390,TAS3siRNA-|ARF4,TAS3siRNA-|ETT,AGO1_miR165-|REV,AGO7_miR390->TAS3siRNA,GTE6->AS1,IPT5->CKh}
5  #Hypothetical regulations
6  hypothetical={AGO10-|AGO1,ANT->FIL,ARF4->FIL,ETT->FIL,FIL->ARF4,FIL->ETT,FIL->KAN1,REV->AGO7,REV->AGO10,REV->IPT5,TAS3siRNA-|miR165,AS1_AS2-|miR165,AS1_AS2-|KAN1,AUXINh->AUXINh,AUXINh->miR390,AUXINh->ANT,AUXINh->ARF4,AUXINh->REV,CKh->GTE6}
7  #Stable states for wild type following the provided order
8  fixed-points()={100110011111001001000,011101100001110111111}
9  #options
10 allow.ambiguity = false
11 allow.additional.states = true
12 allow.additional.cycles = true
13 allow.hypotheses = true
14 block.steady.a.posteriori = false
15 divide.query.by.topology = true
16 topology.iterator.type = radial
17 topological.distance.radius = 10
```

Listing 3: *Griffin* query file representing the constraint of known regulatory interactions, known set of fixed point attractors and 19 hypothetical regulations for the sepal model. The model includes 21 genes (line 2). The set of known and hypothetical regulations, depicted in Fig. 4b in the main text, are specified in lines 4 and 6 respectively. The desired fixed point attractors are defined in line 8. Following (La Rota et al., 2011), we exclude ambiguous regulations (line 10), allow additional attractors (lines 11 and 12) and activate the set of hypotheses (line 13). Additionally, we tell *Griffin* to split the query in multiple queries using the center-radius strategy (lines 15 and 16). In this example the exploration radius is set to 10 (line 17).

```
1  # Name and order of nodes
2  genes={ACR4,AUX_IAA,AUXIN,CLE40,JKD,MGP,MR165,PHB,SCR,SHR,WOX5}
3  # Generalized Interactions,
4  # See Azpeitia et al. (2013)
5  # Single-cell and coupled grn models of cell patterning ...
6  # BMC systems biology, 4(1):1.
7  interactions ={MNU[ACR4:WOX5][AUX_IAA:WOX5][AUXIN:AUX_IAA][CLE40:WOX5][MGP:MGP][MR165:PHB][PHB:JKD][PHB:WOX5],MPU[CLE40:ACR4][JKD:MGP][JKD:SCR][JKD:JKD][SCR:JKD][SCR:MGP][SCR:MR165][SCR:SCR][SHR:JKD][SHR:MGP][SHR:MR165][SHR:SCR],OUSU[ACR4:PHB][AUX_IAA:AUX_IAA][AUX_IAA:PHB][AUX_IAA:SCR][AUXIN:PHB][AUXIN:WOX5][CLE40:PHB][MGP:JKD][MGP:SCR][MGP:SHR][MGP:WOX5][PHB:MR165][PHB:PHB][PHB:SCR][PHB:SHR][SCR:AUXIN][SCR:SHR][SHR:AUX_IAA][SHR:CLE40][SHR:PHB][WOX5:AUX_IAA][WOX5:AUXIN][WOX5:CLE40][WOX5:MGP][WOX5:PHB][WOX5:WOX5],OPU[AUXIN:AUXIN][CLE40:CLE40][MR165:MR165][SHR:SHR]}
8  # Wild type steady states
9  fixed-points()={01000001010,01000010010,01001110110,01001010000,10110000000,00100010010,00101110110,10110010000,00101010111}
10 # Options
11 allow.ambiguity = false
12 allow.additional.states = false
13 allow.additional.cycles = false
14 allow.hypotheses = true
15 block.steady.a.posteriori = true
16 divide.query.by.topology = false
17 limit.boolean.networks = 1
```

Listing 4: *Griffin* query for representing known and hypothetical regulations of the model of Azpeitia et al. (2013). The eleven genes and their ordering are specified at line 2. In the query there are both known and hypothetical interactions, some of the interactions are of unknown sign (for a depiction of the R-regulations see Fig. 4c in the main text). Line 7 defines four groups of R-regulations (for a full list see Table 1 in the main text) a) there are eight interactions of type *MNU*, that is, known (mandatory) interactions of negative sign, b) twelve interactions of type *MPU*, that is, (mandatory) interactions of positive sign, c) twenty six interactions of type *OUSU*, that is, they are hypotheses (it is not known whether they should be in place), for which their sign is not known, and finally d) there are four interactions of type *OPU*, that is, hypothetical regulations of positive sign. In all cases the satisfying interactions are to be unambiguous. The syntax for expressing an interaction is as follows: `[source:target]`, where `source` is the regulating gene and `target` the regulated gene. Line 9 specifies a set of nine known fixed-point attractors. We want all satisfying networks to have this exact set (line 12). No cyclic attractors are allowed (line 13). The strategy for blocking additional fixed-point attractors is performed *a posteriori* (line 15). Finally, as we are only concerned with the existence of any satisfying model, we ask *Griffin* to stop the search after finding the first witness (line 17).

```
1  # Name and order of nodes
2  genes={ACR4,AUX_IAA,AUXIN,CLE40,JKD,MGP,MR165,PHB,SCR,SHR,WOX5}
3  # Generalized Interactions,
4  # See Azpeitia et al. (2013)
5  interactions ={MNU[ACR4:WOX5][AUX_IAA:WOX5][AUXIN:AUX_IAA][CLE40:WOX5][MGP:
      MGP][MR165:PHB][PHB:JKD][PHB:WOX5],MPU[CLE40:ACR4][JKD:MGP][JKD:SCR][JKD:
      JKD][SCR:JKD][SCR:MGP][SCR:MR165][SCR:SCR][SHR:JKD][SHR:MGP][SHR:MR165][
      SHR:SCR],OUSU[ACR4:PHB][AUX_IAA:AUX_IAA][AUX_IAA:PHB][AUX_IAA:SCR][AUXIN:
      PHB][AUXIN:WOX5][CLE40:PHB][MGP:JKD][MGP:SCR][MGP:SHR][MGP:WOX5][PHB:
      MR165][PHB:PHB][PHB:SCR][PHB:SHR][SCR:AUXIN][SCR:SHR][SHR:AUX_IAA][SHR:
      CLE40][SHR:PHB][WOX5:AUX_IAA][WOX5:AUXIN][WOX5:CLE40][WOX5:MGP][WOX5:PHB]
      [WOX5:WOX5],OPU[AUXIN:AUXIN][CLE40:CLE40][MR165:MR165][SHR:SHR]}
6  # Wild type steady states
7  fixed-points()={0**00001010,00100001010,~00000001010,~01100001010,
      0**00010010,00100010010,~00000010010,~01100010010,0**01110110,00101110110
      ,~00001110110,~01101110110,0**0*010000,0010*010000,~0000*010000,
      ~0110*010000,1**10000000,10110000000,~10010000000,~11110000000,
      1***0010000,101*0010000,~100*0010000,~111*0010000,0**01010111,00101010111
      ,~00001010111,~01101010111}
8  # Options
9  allow.ambiguity = false
10 allow.additional.states = false
11 allow.additional.cycles = false
12 allow.hypotheses = true
13 block.steady.a.posteriori = true
14 divide.query.by.topology = false
15 limit.boolean.networks = 1
```

Listing 5: *Griffin* query for representing known and hypothetical regulations of the *A. thaliana* root model with uncertainty in the set of fixed point attractors. Lines are the same as in Listing 4, with the exception of line 7. It defines a combination of constraints on partially known fixed point attractors and prohibitions to express uncertainty in the value of some of the genes. For a full explanation on the meaning of the fixed point attractor constraints of line 7 see Table S1.

```
# Name and order of nodes
genes={ACR4,AUX_IAA,AUXIN,CLE40,JKD,MGP,MR165,PHB,SCR,SHR,WOX5}
# Generalized Interactions
# See Azpeitia et al. (2013)
interactions ={MNU[ACR4:WOX5][AUX_IAA:WOX5][AUXIN:AUX_IAA][CLE40:WOX5][MGP:MGP][MR165:PHB][PHB:JKD][PHB:WOX5],MPU[CLE40:ACR4][JKD:MGP][JKD:SCR][JKD:JKD][SCR:JKD][SCR:MGP][SCR:MR165][SCR:SCR][SHR:JKD][SHR:MGP][SHR:MR165][SHR:SCR],OUSU[ACR4:ACR4][ACR4:AUX_IAA][ACR4:AUXIN][ACR4:JKD][ACR4:MGP][ACR4:PHB][ACR4:SCR][ACR4:SHR][AUX_IAA:AUX_IAA][AUX_IAA:PHB][AUX_IAA:SCR][AUXIN:PHB][AUXIN:WOX5][CLE40:AUX_IAA][CLE40:AUXIN][CLE40:JKD][CLE40:MGP][CLE40:PHB][CLE40:SCR][CLE40:SHR][JKD:ACR4][JKD:AUX_IAA][JKD:AUXIN][JKD:CLE40][JKD:PHB][JKD:SHR][JKD:WOX5][MGP:ACR4][MGP:AUX_IAA][MGP:AUXIN][MGP:CLE40][MGP:JKD][MGP:PHB][MGP:SCR][MGP:SHR][MGP:WOX5][PHB:ACR4][PHB:AUX_IAA][PHB:AUXIN][PHB:CLE40][PHB:MGP][PHB:MR165][PHB:PHB][PHB:SCR][PHB:SHR][SCR:ACR4][SCR:AUX_IAA][SCR:AUXIN][SCR:CLE40][SCR:PHB][SCR:SHR][SHR:ACR4][SHR:AUX_IAA][SHR:AUXIN][SHR:CLE40][SHR:PHB][WOX5:ACR4][WOX5:AUX_IAA][WOX5:AUXIN][WOX5:CLE40][WOX5:JKD][WOX5:MGP][WOX5:PHB][WOX5:SCR][WOX5:SHR][WOX5:WOX5],OPU[AUXIN:AUXIN][CLE40:CLE40][MR165:MR165][SHR:SHR]}
# Wild type steady states
fixed-points()={0**00001010,00100001010,~00000001010,~01100001010,0**00010010,00100010010,~00000010010,~01100010010,0**01110110,00101110110,~00001110110,~01101110110,0**0*010000,0010*010000,~0000*010000,~0110*010000,1**10000000,10110000000,~10010000000,~11110000000,1***0010000,101*0010000,~100*0010000,~111*0010000,0**01010111,00101010111,~00001010111,~01101010111}
# Options
allow.ambiguity = false
allow.additional.states = false
allow.additional.cycles = false
allow.hypotheses = true
block.steady.a.posteriori = true
divide.query.by.topology = false
limit.boolean.networks = 1
```

Listing 6: *Griffin* query for representing known and hypothetical regulations of the modified model of Azpeitia et al. (2013).

2 PARTIALLY DEFINED FIXED-POINT ATTRACTORS AND EXPLICIT EXCLUSIONS

This section includes a detailed explanation on the syntax expressing partially defined fixed-point attractors and explicit exclusions for the query given in Listing 6.

No.	Constraint	Concrete states		Decimal notation	
1	0**00001010	00000001010<br>01000001010	00100001010<br>01100001010	~~10~~<br>522	266<br>~~778~~
2	00100001010	00100001010		266	
3	∼00000001010	00000001010		~~10~~	
4	∼01100001010	01100001010		~~778~~	
5	0**00010010	00000010010<br>01000010010	00100010010<br>01100010010	~~18~~<br>530	274<br>~~786~~
6	00100010010	00100010010		274	
7	∼00000010010	00000010010		~~18~~	
8	∼01100010010	01100010010		~~786~~	
9	0**01110110	00001110110<br>01001110110	00101110110<br>01101110110	~~118~~<br>630	374<br>~~886~~
10	00101110110	00101110110		374	
11	∼00001110110	00001110110		~~118~~	
12	∼01101110110	01101110110		~~886~~	
13	0**0*010000	00000010000<br>00100010000<br>01000010000<br>01100010000	00001010000<br>00101010000<br>01001010000<br>01101010000	~~16~~<br>272<br>528<br>~~784~~	~~80~~<br>336<br>592<br>~~848~~
14	0010*010000	00100010000	00101010000	272	336
15	∼0000*010000	00000010000	00001010000	~~16~~	~~80~~
16	∼0110*010000	01100010000	01101010000	~~784~~	~~848~~
17	1**10000000	10010000000<br>11010000000	10110000000<br>11110000000	~~1152~~<br>1664	1408<br>~~1920~~
18	10110000000	10110000000		1408	
19	∼10010000000	10010000000		~~1152~~	
20	∼11110000000	11110000000		~~1920~~	
21	1***0010000	10000010000<br>10100010000<br>11000010000<br>11100010000	10010010000<br>10110010000<br>11010010000<br>11110010000	~~1040~~<br>1296<br>1552<br>~~1808~~	~~1168~~<br>1424<br>1680<br>~~1936~~
22	101*0010000	10100010000	10110010000	1296	1424
23	∼100*0010000	10000010000	10010010000	~~1040~~	~~1168~~
24	∼111*0010000	11100010000	11110010000	~~1808~~	~~1936~~
25	0**01010111	00001010111<br>01001010111	00101010111<br>01101010111	~~87~~<br>599	343<br>~~855~~
26	00101010111	00101010111		343	
27	∼00001010111	00001010111		~~87~~	
28	∼01101010111	01101010111		~~855~~	

Table S1: Encoding of partial knowledge in the fixed-point attractors of *A. thaliana* root model. The constraint column lists all fixed-point attractor constraints. Concrete states are the simplest of constraints, they are specified at rows 2, 6, 10, 18 and 26, they require the mentioned state to be present as a fixed-point attractor in the dynamics of satisfying Boolean networks. The rows that include states with wildcard characters (*) indicate uncetarinty about the corresponding gene activation state. For example, following the ordering defined in Listing 5, the constraint 0**00001010 in row 2, defines uncertainty in the state of genes *AUX_IAA* and *AUXIN*, this partially known fixed-point attractor describes four concrete states listed in the third column (resulting from all possible combinations of gene states at the wildcard locations). To facilitate the explanation, the fourth column represents the states as integer numbers. Other partially known fixed-point attractors are listed in rows 5, 9, 13, 14, 17, 21, 22 and 25. *Griffin* ensures that satisfying Boolean networks will exhibit fixed-point attractors corresponding with at least one of the possible concrete states for each of the listed partially known fixed-point attractors. *Griffin* allows for further refinement of the uncertainty by allowing to prohibit specific fixed-point attractors. To exclude a particular fixed-point attractor from appearing in a satisfying solution, we simply precede the state with a tilde ($\sim$). For example, although the partially known fixed-point attractor mentioned at row 5 asks the Boolean network to have at least any of the fixed-point attractors in the set $\{18, 274, 530, 786\}$, the prohibitions at rows 7 and 8 will exclude fixed-point attractors 18 and 786, respectively. Further prohibitions of this type are listed at rows 3, 4, 11, 12, 19, 20, 27 and 28. Prohibitions can be defined in partially known fixed-point attractors such as those in rows 15, 16, 23 and 24. In this case all possible concrete states in the prohibition are excluded from the answer. The fourth column shows prohibitions as crossed out states, indicating they are not to appear as fixed-point attractors of satisfying networks. If a state appears on its own in the last column it means that it will be unconditionally present as a fixed-point attractor of any satisfying solution (all unconditional states are colored green). If there is more than one not crossed out state in any particular row of the table, it means that those states are optional, and may or may not appear as fixed-point attractors in any satisfying network (optional states are colored blue). In total, we are asking for five unconditional (always present) fixed-point attractors: 266, 274, 343, 374 and 1408; and thirteen optional fixed-point attractor: 272, 336, 522, 528, 530, 592, 599, 630, 1296, 1424, 1552, 1664 and 1680. Note that optional states are conditioned to certain properties according to the particular combination of constraints expressed. For example, the optional fixed-point attractors at row 13 cannot simultaneously be all missing, at least one of them must be present in the solution. fixed-point attractors in lines 14, 17, 21 and 22 are subjected to the same requirement.

REFERENCES

Alvarez-Buylla, E. R., Benítez, M., Corvera-Poiré, A., Chaos Cador, Á., de Folter, S., Gamboa de Buen, A., et al. (2010). Flower development. *The Arabidopsis Book* , e0127doi:10.1199/tab.0127

Azpeitia, E., Weinstein, N., Benítez, M., Mendoza, L., and Alvarez-Buylla, E. R. (2013). Finding missing interactions of the *Arabidopsis thaliana* root stem cell niche gene regulatory network. *Frontiers in Plant Science* 4. doi:10.3389/fpls.2013.00110

La Rota, C., Chopard, J., Das, P., Paindavoine, S., Rozier, F., Farcot, E., et al. (2011). A data-driven integrative model of sepal primordium polarity in arabidopsis. *The Plant Cell Online* 23, 4318–4333. doi:10.1105/tpc.111.092619
